# A morphological and molecular study of *Hydrodynastes gigas* (Serpentes, Dipsadidae), a widespread species from South America

**DOI:** 10.7717/peerj.10073

**Published:** 2020-11-25

**Authors:** Priscila S. Carvalho, Hussam Zaher, Nelson J. da Silva Jr, Diego J. Santana

**Affiliations:** 1 Instituto de Biociências, Universidade Federal de Mato Grosso do Sul, Campo Grande, Mato Grosso do Sul, Brazil; 2 Instituto de Biociências, Letras e Ciências Exatas, Universidade Estadual Paulista, São José do Rio preto, São Paulo, Brazil; 3 Museu de Zoologia da Universidade de São Paulo, São Paulo, São Paulo, Brazil; 4 Escola de Ciências Médicas, Farmacêuticas e Biomédicas, Pontifícia Universidade Católica de Goiás, Goiânia, Goiás, Brazil

**Keywords:** Biodiversity, Integrative taxonomy, Melanism, Neotropical region, Polymorphism, Squamata, Synonymization, Xenodontinae, Watersheds

## Abstract

**Background:**

Studies with integrative approaches (based on different lines of evidence) are fundamental for understanding the diversity of organisms. Different data sources can improve the understanding of the taxonomy and evolution of snakes. We used this integrative approach to verify the taxonomic status of *Hydrodynastes gigas* (Duméril, Bibron & Duméril, 1854), given its wide distribution throughout South America, including the validity of the recently described *Hydrodynastes melanogigas* Franco, Fernandes & Bentim, 2007.

**Methods:**

We performed a phylogenetic analysis of Bayesian Inference with mtDNA 16S and Cytb, and nuDNA Cmos and NT3 concatenated (1,902 bp). In addition, we performed traditional morphometric analyses, meristic, hemipenis morphology and coloration pattern of *H*. *gigas* and *H*. *melanogigas*.

**Results:**

According to molecular and morphological characters, *H. gigas* is widely distributed throughout South America. We found no evidence to support that *H. gigas* and *H. melanogigas* species are distinct lineages, therefore, *H. melanogigas* is a junior synonym of *H. gigas*. Thus, the melanic pattern of *H. melanogigas* is the result of a polymorphism of *H. gigas*. Melanic populations of *H. gigas* can be found in the Tocantins-Araguaia basin.

## Introduction

Species are considered lineages with distinct evolutionary histories (De Queiroz, 2007). Taxonomic studies are traditionally based on morphological descriptors to delimit species (e.g.,  Franco et al., 2017; França et al., 2018; Meneses-Pelayo & Passos, 2019). However, in many cases, species are difficult to delimit due to the limited number of morphological differences or the absence of them, preventing the recognition of valid cryptic species (Bickford et al., 2007). Morphology alone might result in more than one name being assigned to individuals belonging to the same evolutionary lineage (i.e., species) (Passos & Prudente, 2012; Passos, Martins & Pinto-Coelho, 2016; Mângia et al., 2020). Many species are described based solely on morphological patterns, which could merely reflect interpopulational variation, instead of evidence of lineage separation (e.g.,  Brusquetti et al., 2014; Mângia et al., 2020).

Currently, species delimitation must be based on the integration of more than one type of data set (e.g., DNA sequences, morphology, behavior, pheromone), which helps improve taxonomic understanding (Dayrat, 2005; Padial & De La Riva, 2010; Padial et al., 2010; Pante, Schoelinck & Puillandre, 2014). An integrative approach contributes to taxonomic, phylogenetic and phylogeographic studies (Pante, Schoelinck & Puillandre, 2014), being thus useful in delimiting species and sorting possible interspecific variations and lineages with similar morphologies. Ultimately, integrative approaches are essential for testing taxonomic schemes and correcting nomenclatural inconsistencies (e.g.,  Recoder et al., 2014; Ruane et al., 2018; Mângia et al., 2020).

Among the morphological characteristics adopted in taxonomic studies that usually result in inaccurate nomenclatural decisions are coloration pattern variations (e.g., *Atractus* spp.: Passos, Martins & Pinto-Coelho, 2016; *Apostolepis* spp.: Entiauspe-Neto et al., 2019). Animal coloration has several adaptive functions, including thermoregulation, signaling and protection (Briolat et al., 2019). Variation in coloration patterns (polychromatism), often associated with ontogenetic dimorphism (e.g., *Corallus* spp.: Henderson, 1997; Henderson, Passos & Feitosa, 2009; and *Drymoluber* spp.: Costa, Moura & Feio, 2013) or with chromatic anomalies such as leukism, albinism and melanism (the latter caused by the increase of epidermal pigments known as melanin; Kettlewell, 1973) can lead to erroneous nomenclatural decisions. Although there are some exclusively melanistic species (e.g., some species of the Pseudoboini tribe), melanism can also be the result of intraspecific polymorphism (e.g.,  Bernardo et al., 2012). The adaptive value of melanism may be related to predation, protection or thermoregulation (Andrén & Nilson, 1981; Forsman & Ås, 1987; Capula, Luiselli & Monney, 1995; Briolat et al., 2019).

*Hydrodynastes* Fitzinger, 1843 is a genus of large semiaquatic snakes, which currently contains three species: *Hydrodynastes bicinctus* (Hermann, 1804) distributed in Colombia, Venezuela, French Guiana, Guyana, Suriname and Brazil (Murta-Fonseca, Franco & Fernandes, 2015); *Hydrodynastes gigas* (Duméril, Bibron & Duméril, 1854) distributed in French Guiana, Bolivia, Paraguay, Argentina and Brazil (Giraudo & Scrocchi, 2002; Pereira-Filho & Montingelli, 2006; Wallach, Williams & Boundy, 2014; Nogueira et al., 2019); and *Hydrodynastes melanogigas* (Franco, Fernandes & Bentim, 2007) recorded only in the Tocantins-Araguaia Basin in the states of Tocantins, Mato Grosso and Maranhão, Brazil (Silva Jr et al., 2012; Santos Jr et al., 2017). *Hydrodynastes gigas* (Duméril, Bibron & Duméril, 1854) has a wide distribution throughout Brazil, and has been recorded in the states of Amapá, Amazonas, Pará, Rondônia, Roraima, Tocantins, Maranhão, Piauí, Rio Grande do Norte, Mato Grosso, Mato Grosso do Sul, Minas Gerais, São Paulo, Paraná, Rio Grande do Sul (Nogueira et al., 2019).

Given the wide distribution of *Hydrodynastes gigas* in South America, a level of intraspecific variation throughout its populations is to be expected, with some potential to represent still undescribed cryptic species. Indeed, Franco, Fernandes & Bentim (2007) considered one of these populations as a distinct species, describing *H. melanogigas* mainly through its differential color pattern and pointed out its similarity with *H. gigas* on meristic and hemipenial characteristics. Therefore, the distinction between *H. melanogigas* and *H. gigas* rests mainly on its melanistic color pattern and on its inferred allopatric distribution with *H. gigas*. The present study aims to evaluate the taxonomic validity of *Hydrodynastes gigas* and *Hydrodynastes melanogigas* using an integrative taxonomic approach inferred by molecular and morphological data.

## Materials & Methods

We evaluated the taxonomic status of *Hydrodynastes gigas and Hydrodynastes melanogigas* by sequencing two mitochondrial and two nuclear genes for 32 individuals belonging to the two species. We further analyzed the external morphology of 186 specimens of *H. gigas and H. melanogigas*.

### Molecular analysis

**Figure 1:**
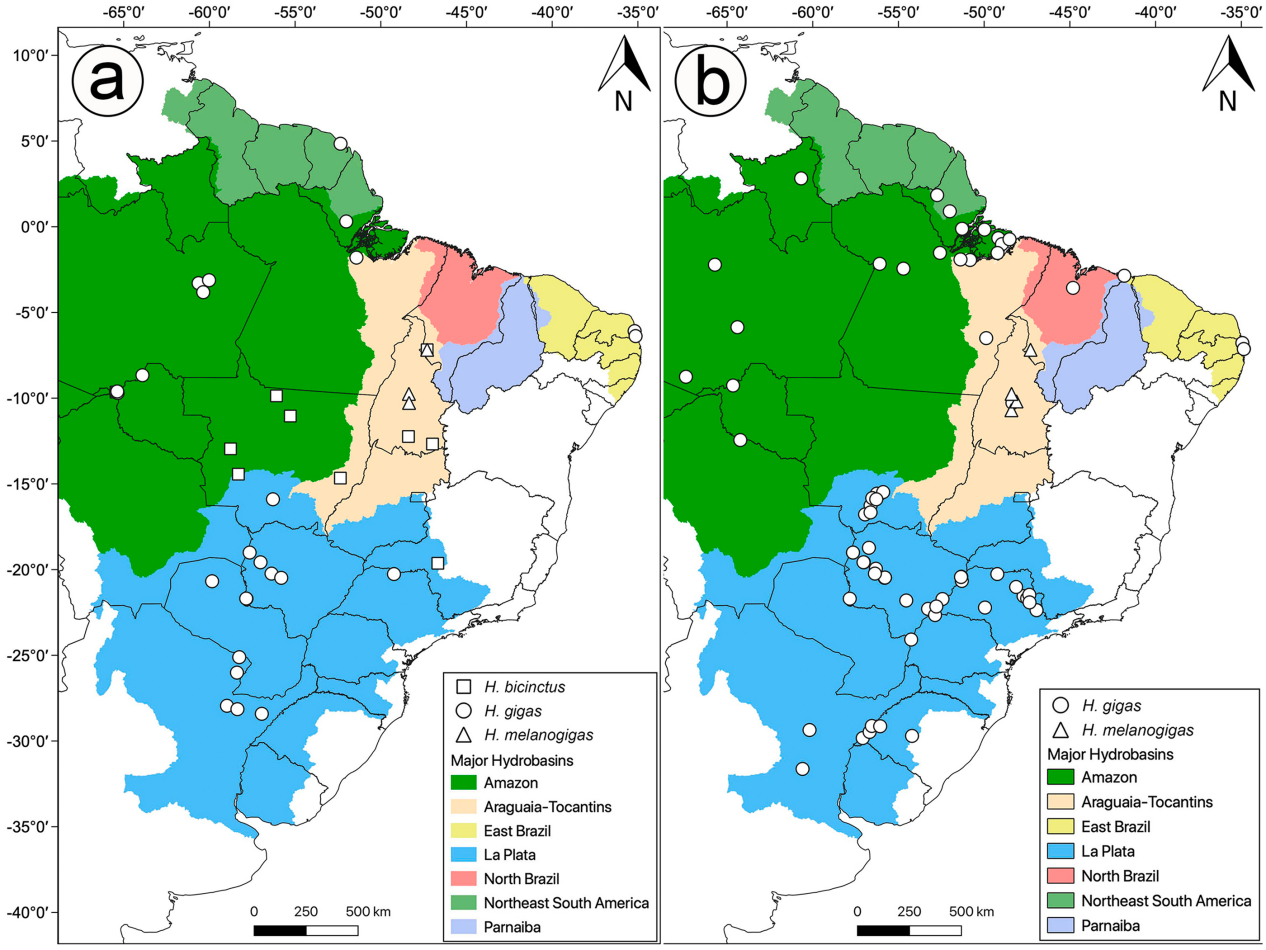
Distribution of analyzed data in this study. Sample localities for (A) molecular and (B) morphology of *Hydrodynastes* analyzed in this study.

We extracted the DNA from muscle, liver or scale of 27 samples of *Hydrodynastes gigas,* five of *H. melanogigas* and 12 of *H. bicinctus*. Samples of *Hydrodynastes bicinctus* were added to our analysis in order to provide a complete species representation for the genus. We used the phenol-chloroform extraction protocol (Sambrook, Fritsch & Maniatis, 1989) (Fig. 1A). We amplified the partial sequences of the mitochondrial 16S ribosomal (mtDNA) genes (16S rRNA, 326 pb) (Palumbi et al., 2002), Cytochrome b (Cytb, 618 pb) (Pook, Wüster & Thorpe, 2000), the nuclear genes (nuDNA) Oocyte maturation factor Mos (Cmos, 478 pb) (Lawson et al., 2005) and Neurotrophin-3 (NT3, 480 pb) (Townsend et al., 2008) using the standard Polymerase Chain Reaction (PCR) technique as described by (Pook, Wüster & Thorpe, 2000) and Moraes-Da-Silva et al. (2019). We visually checked all nucleotide sequences and aligned the concatenated genes (1,902 pb) using the Muscle algorithm (Edgar, 2004) in the Geneious v.9.0.5 program (https://www.geneious.com). We used the species *Pseudoboa nigra* and *Xenopholis scalaris* as outgroup of *Hydrodynastes* (Vidal, Dewynter & Gower, 2010; Zaher et al., 2009), and rooted the tree in *Xenopholis*. We used the sequences available in GenBank and deposited those generated in this study into the same database (Table 1).

We used PartitionFinder 2 to identify partitioning schemes and the most appropriate nucleotide replacement models (Lanfear et al., 2016). According to our concatenated alignment, we found five partitions evaluated by BIC (Table 2). For phylogenetic analysis, we used the Bayesian inference implemented in MrBayes v3.2.6 (Ronquist & Huelsenbeck, 2003) using the substitution models generated by PartitionFinder. We ran two independent runs of four Markov chains for 20 million generations sampling every 5,000 generations and discarding 25% as burn-in. We evaluated the stability of the analysis in Tracer v1.6, ensuring that all ESS values were above 200 (Rambaut et al., 2014). We calculated the divergence between sequences (p-distance) in Mega v10.0.5 (Tamura et al., 2013).

**Table 1:** Voucher information and GenBank numbers. Specimens used for the molecular analyses, including GenBank numbers for mitochondrial 16S and Cytb, nuclear Cmos and NT3 sequences. * data not available in the original references.

**Species**	**Voucher**	**Locality**	**Genbank Accession number**				**Reference**
			**16S**	**Cytb**	**Cmos**	**NT3**	
*Hydrodynastes bicinctus*	CHUNB52057	Brazil, Maranhão, Carolina	MT192271	MT224977	MT328069	MT328103	This study
*Hydrodynastes bicinctus*	CHUNB47129	Brazil, Mato Grosso, Alta Floresta	MT192270	MT224976	–	–	This study
*Hydrodynastes bicinctus*	CHUNB63637	Brazil, Mato Grosso, Nova Xavantina	MT192272	MT224978	MT328070	MT328104	This study
*Hydrodynastes bicinctus*	MZUSP17831	Brazil, Mato Grosso, Itaúba	MT192274	MT224980	MT328072	MT328105	This study
*Hydrodynastes bicinctus*	UFMT7550	Brazil, Mato Grosso, Tangará da Serra	MT192276	MT224981	MT328073	MT328107	This study
*Hydrodynastes bicinctus*	UFMT7551	Brazil, Mato Grosso, Tangará da Serra	MT192277	MT224982	MT328074	MT328108	This study
*Hydrodynastes bicinctus*	1817 (MZUSP)	Brazil, Mato Grosso, Sapezal	MT192267	MT224972	MT328065	MT328100	This study
*Hydrodynastes bicinctus*	MZUSP20580	Brazil, Rondônia, Porto Velho, Mutum	MT192275	–	–	MT328106	This study
*Hydrodynastes bicinctus*	CHUNB38982	Brazil, Tocantins, Arraias	MT192268	MT224973	MT328066	–	This study
*Hydrodynastes bicinctus*	CHUNB40618	Brazil, Tocantins, Mateiros	–	MT224974	MT328067	MT328101	This study
*Hydrodynastes bicinctus*	CHUNB40619	Brazil, Tocantins, Mateiros	MT192269	MT224975	MT328068	MT328102	This study
*Hydrodynastes bicinctus*	MZUSP15560	Brazil, Tocantins, UHE Peixe Angical	MT192273	MT224979	MT328071	–	This study
*Hydrodynastes gigas*	INALI6573	Argentina, Chaco, San Fernando	MT192283	MT224989	–	MT328114	This study
*Hydrodynastes gigas*	INALI4779	Argentina, Corrientes, General San Martín	MT192282	MT224988	–	MT328113	This study
*Hydrodynastes gigas*	INALI6867	Argentina, Corrientes, Mburucuya	MT192285	MT224991	–	MT328116	This study
*Hydrodynastes gigas*	LGE7992	Argentina, Formosa, Pirané	MT192287	MT224995	–	MT328119	This study
*Hydrodynastes gigas*	INALI6731	Argentina, Formosa, Pilcomayo	MT192284	MT224990	–	MT328115	This study
*Hydrodynastes gigas*	MZUSP11704	Brazil, Amapá	MT192289	MT225000	–	MT328124	This study
*Hydrodynastes gigas*	INPA-HT5513	Brazil, Amazonas, Careiro	MT215328	MT224994	MT328081	MT328118	This study
*Hydrodynastes gigas*	INPA-HT190	Brazil, Amazonas, Manaus	MT215327	MT224992	MT328079	MT328117	This study
*Hydrodynastes gigas*	MTR19444	Brazil, Amazonas, Manacapuru	MT215333	MT224999	MT328086	MT328123	This study
*Hydrodynastes gigas*	CHUNB65028	Brazil, Mato Grosso, Nossa Senhora do Livramento	MT192281	MT224986	MT328077	MT328111	This study
*Hydrodynastes gigas*	MAP-T3894	Brazil, Mato Grosso do Sul, Porto Murtinho	MT215330	MT224997	MT328083	MT328121	This study
*Hydrodynastes gigas*	MAP4050	Brazil, Mato Grosso do Sul, Porto Murtinho	MT215329	MT224996	MT328082	MT328120	This study
*Hydrodynastes gigas*	ZUFMS-REP2392	Brazil, Mato Grosso do Sul, Anastácio	MT192296	MT225007	MT328092	MT328130	This study
*Hydrodynastes gigas*	ZUFMS-REP 2393	Brazil, Mato Grosso do Sul, Corumbá	MT192297	MT225008	MT328093	MT328131	This study
*Hydrodynastes gigas*	ZUFMS-REP 2395	Brazil, Mato Grosso do Sul, Miranda	MT192298	MT225009	MT328094	MT328132	This study
*Hydrodynastes gigas*	ZUFMS-REP 2476	Brazil, Mato Grosso do Sul, Corumbá	MT192299	MT225010	MT328095	MT328133	This study
*Hydrodynastes gigas*	ZUFMS-REP 2389	Brazil, Minas Gerais, Fronteira	MT192295	MT225006	MT328091	MT328129	This study
*Hydrodynastes gigas*	MPEG21864	Brazil, Pará, Melgaço	MT192288	MT224998	MT328084	MT328122	This study
*Hydrodynastes gigas*	AAGARDA8745	Brazil, Rio Grande do Norte, Nísia Floresta	MT192278	MT224983	MT328075	MT328109	This study
*Hydrodynastes gigas*	AAGARDA12357	Brazil, Rio Grande do Norte, Canguaretama	MT192279	MT224984	–	–	This study
*Hydrodynastes gigas*	INPA-HT5427	Brazil, Rondônia, Porto Velho, Teotônio	MT192286	MT224993	MT328080	–	This study
*Hydrodynastes gigas*	MZUSP18572	Brazil, Rondônia, Porto Velho, Mutum	MT192290	MT225001	MT328087	MT328125	This study
*Hydrodynastes gigas*	MZUSP18573	Brazil, Rondônia, Porto Velho, Abunã	MT192291	MT225002	MT328088	MT328126	This study
*Hydrodynastes gigas*	MZUSP19710	Brazil, Rondônia, Porto Velho, Abunã	MT192292	MT225003	MT328089	MT328127	This study
*Hydrodynastes gigas*	MZUSP20449	Brazil, Rondônia, Porto Velho, Abunã	MT192293	MT225004	MT328090	MT328128	This study
*Hydrodynastes gigas*	AF2382	French Guiana, Matoury	MT192280	MT224985	MT328076	MT328110	This study
*Hydrodynastes gigas*	PINV1580254	Paraguay, Alto Paraguay	MT192294	MT225005	–	MT424769	This study
*Hydrodynastes melanogigas*	MPEG24383	Brazil, Maranhão, Carolina	MT215331	MT225012	–	MT328097	This study
*Hydrodynastes melanogigas*	MPEG24384	Brazil, Maranhão, Carolina	MT215332	MT225013	MT328085	MT328098	This study
*Hydrodynastes melanogigas*	MZUSP19557	Brazil, Maranhão, Carolina	MT215334	MT225014	–	MT328099	This study
*Hydrodynastes melanogigas*	IBSP65144	Brazil, Tocantins, Lajeado	–	MT224987	MT328078	MT328112	This study
*Hydrodynastes melanogigas*	UFMS-REP3446	Brazil, Tocantins, Palmas	MT215335	MT225011	MT328096	MT328134	This study
*Pseudoboa nigra*	MZUSP13278	*	GQ457764	JQ598948	GQ457885	–	Zaher et al. (2009) and Grazziotin et al. (2012)
*Xenopholis scalaris*	KU222204	*	JQ598915	GQ895897	JQ599002	–	Pyron et al. (2011) and Grazziotin et al. (2012)

**Table 2:** PartitionFinder 2 model of nucleotide substitution. Best-fitting partitioning scheme model of nucleotide substitution for 16S, Cytb, Cmos and NT3 genes.

Partitioning scheme	Model
Cytb1, 16S	GTR+G
Cytb2, Cmos2	HKY+I
Cytb3	HKY+G
NT32, Cmos3, Cmos1	JC
NT31, NT33	K80+I+G

### Morphological analysis

We examined 144 specimens of *Hydrodynastes gigas* and 42 specimens of *H. melanogigas* (Fig. 1B). Museum acronyms follow Sabaj-Pérez (2014), except for Coleção Herpetológica da Universidade Federal da Paraíba (CHUFPB), João Pessoa, PB; Coleção Zoológica Delta do Parnaíba (CZDP), Parnaíba, PI; Universidade Luterana do Brasil (MZCEULP), Palmas, TO. The specimens examined are listed in the Appendix S1.

Franco, Fernandes & Bentim (2007) described *Hydrodynastes melanogigas* based on 17 specimens collected in the municipalities of Palmas (type locality), Porto Nacional, and Lajeado, which are all located in the state of Tocantins, Brazil. Unfortunately, most of the type series was lost in the 2010 fire that occurred at the Instituto Butantan. Currently, only three individuals remain from the type series: two at the proper Institute in São Paulo (paratypes IBSP 65978 and IBSP 66387) and one at the Museu Nacional do Rio de Janeiro (paratype MNRJ 15101). From the remaining type-series, we analyzed all the remaining individuals.

We examined 14 meristic characters (Table 3) and eight morphometric ones (Table 4), in addition to the coloration pattern and morphology of the hemipenis. Sex was determined by the presence or absence of hemipenes through a ventral incision at the base of the tail. We measured individuals with an electronic caliper (0.01 mm) and a flexible ruler (1 mm), on their right side whenever possible. In order to test morphometric differences between *H. gigas* and *H. melanogigas*, we conducted a principal component analysis (PCA) and took the first two principal components of the ordination to create a MANOVA. We ran this analysis with adult males and females separately, and performed the analysis in R software (R Core Team, 2014) using the package Vegan (Oksanen et al., 2007). We followed the terminology of Dowling (1951) for counting the ventral scales, and Peters (1964) and Vanzolini, Ramos-Costa & Vitt (1980) for pholidosis. We surveyed the geographic coordinates of the data catalogs of zoological collections using Google Earth software*.*

**Table 3:** Meristic characters in *Hydrodynastes gigas* and *Hydrodynastes melanogigas*.

Variables	*Hydrodynastes gigas*			*Hydrodynastes melanogigas*	
	Male	Female	Undetermined^**^	Male	Female
SLl	8 (*n* = 64); 9 (*n* = 1)	8 (*n* = 61); 9 (*n* = 5)	8 (*n* = 6)	8 (*n* = 22)	8 (*n* = 17)
ILr	9 (*n* = 5); 10 (*n* = 37); 11 (*n* = 21); 12 (*n* = 1)	9 (*n* = 2); 10 (*n* = 29); 11 (*n* = 31); 12 (*n* = 5)	10 (*n* = 3); 11 (*n* = 2)	8 (*n* = 1); 9 (*n* = 2); 10 (*n* = 17); 11 (*n* = 2)	9 (*n* = 1); 10 (*n* = 14); 11 (*n* = 2)
Ill	9 (*n* = 4); 10 (*n* = 32); 11 (*n* = 26)	9 (*n* = 3); 10 (*n* = 24); 11 (*n* = 35); 12 (*n* = 4)	10 (*n* = 3); 11 (*n* = 3)	9 (*n* = 4); 10 (*n* = 16); 11 (*n* = 2)	10 (*n* = 10); 11 (*n* = 6); 12 (*n* = 1)
LO	1 (*n* = 68)	1 (*n* = 69)	1 (*n* = 7)	1 (*n* = 22)	1 (*n* = 17)
PE	1 (*n* = 66); 2 (*n* = 1)	1 (*n* = 68); 2 (*n* = 1)	1 (*n* = 7)	1 (*n* = 20); 2 (*n* = 2)	1 (*n* = 16); 2 (*n* = 1)
PO	2 (*n* = 48); 3 (19)	1 (*n* = 1); 2 (*n* = 64); 3 (*n* = 4)	2 (*n* = 7)	2 (*n* = 19); 3 (*n* = 3)	2 (*n* = 17)
SO	2 (*n* = 3); 3 (*n* = 64)	2 (*n* = 4); 3 (*n* = 65)	3 (*n* = 7)	3 (*n* = 22)	3 (*n* = 17)
AT	1 (*n* = 5); 2 (60); 3 (*n* = 2)	1 (*n* = 5); 2 (*n* = 62); 3 (*n* = 2)	2 (*n* = 5); 3 (*n* = 1)	2 (*n* = 16); 3 (*n* = 5); 4 (*n* = 1)	2 (*n* = 17)
PT	1+2 (*n* = 5); 1+3 (*n* = 6); 2+1 (*n* = 1); 2+2 (*n* = 3); 2+3 (*n* = 49); 2+4 (*n* = 2); 3+3 (*n* = 2)	2 (*n* = 1); 3 (*n* = 1); 1+2 (*n* = 3); 1+3 (*n* = 9); 2+2 (*n* = 7); 2+3 (*n* = 45); 2+4 (*n* = 1); 3+2 (*n* = 1); 3+3 (*n* = 1)	2+2 (*n* = 2); 2+3 (*n* = 4); 2+4 (*n* = 1)	1+3 (*n* = 3); 2+1 (*n* = 1); 2+2 (*n* = 2); 2+3 (*n* = 15)	1+3 (*n* = 3); 2+2 (*n* = 2); 2+3 (*n* = 12)
NA	2 (*n* = 68)	2 (*n* = 69)	2 (*n* = 7)	2 (*n* = 22)	2 (*n* = 17)
IL+G1	i-iv (*n* = 15); i-v (*n* = 48); i-vi (*n* = 3)	i-ii (*n* = 1); i-iv (*n* = 10); i-v (*n* = 54); i-vi (*n* = 3)	i-iv (*n* = 2); i-v (*n* = 5)	i-iv (*n* = 1); i-v (*n* = 19); i-vi (*n* = 2)	i-v (*n* = 16); i–iv (*n* = 1)
IL+G2	0 (*n* = 2); v-vi (*n* = 7); v (*n* = 8); vi (*n* = 46); vii (*n* = 3)	0 (*n* = 3); v-vi (*n* = 5); v (*n* = 4); vi (*n* = 56); vii (*n* = 1)	v (*n* = 2); vi (*n* = 5)	v (*n* = 1); vi (*n* = 19); vii (*n* = 2)	vi (*n* = 15); v–vi (*n* = 1); vi–vii (*n* = 1)
AP	2 (*n* = 67)	2 (*n* = 69)	2 (*n* = 4)	2 (*n* = 22)	2 (*n* = 17)
AD	17 (*n* = 1); 18 (*n* = 1); 19 (*n* = 59); 20 (*n* = 3); 21 (*n* = 4)	18 (*n* = 3); 19 (*n* = 54); 20 (*n* = 5); 21 (*n* = 6)	19 (*n* = 4)	19 (*n* = 22)	19 (*n* = 17)
MD	17 (*n* = 2); 18 (*n* = 1); 19 (*n* = 64)	17 (*n* = 4); 19 (*n* = 65)	17 (*n* = 1); 19 (*n* = 3)	17 (*n* = 3); 19 (*n* = 19)	18 (*n* = 1); 19 (*n* = 16)
PD	14 (*n* = 1); 15 (*n* = 67)	14 (*n* = 1); 15 (*n* = 67); 17 (*n* = 1)	15 (*n* = 4)	14 (*n* = 3); 15 (*n* = 18); 16 (*n* = 1)	15 (*n* = 17)
PV	1 (*n* = 22); 2 (*n* = 42); 3 (*n* = 4)	1 (*n* = 18); 2 (*n* = 45); 3 (*n* = 6)	1 (*n* = 2); 2 (*n* = 1)	1 (*n* = 10); 2 (*n* = 12)	1 (*n* = 4); 2 (*n* = 13)
VE	150–164	152–169		154–168	168–172
SC^*^	58(9) –88	49(30)–84		71–86	70(24) –79

**Notes.**

In parenthesis the sampled number (n).

SLr right supralabials SLl left supralabials ILr right infralabials Ill left infralabials LO loreal PE preoculars PO postoculars SO suboculars AT anterior temporals PT posterior temporals NA nasal IL+G1 infralabials in contact with first pair of genials IL+G2 infralabials in contact with second pair of genials AP apical pits AD anterior dorsal scale rows MD midbody dorsal scale rows PD posterior dorsal scale rows PV preventrals VE ventral SC subcaudal

* 75 specimens present autotomized tail.

** specimens present only the head or other body part.

**Table 4:** Morphometric measurement in *Hydrodynastes gigas* and *Hydrodynastes melanogigas*.

Variables	*Hydrodynastes gigas*		*Hydrodynastes melanogigas*	
	Male	Female	Male	Female
SVL	249–1747 (*n* = 68) 998 ± 1059,25	277–1879 (*n* = 69) 988.5 ± 512.2	468–1548 (*n* = 22) 1100.4 ± 396.1	524–2198 (*n* = 17) 1048.3 ± 381.4
TL	84 (25)–580 (*n* = 68) 302,5 ± 392,44	71–543 (*n* = 69) 276.6 ± 143.6	180–548 (*n* = 22) 351.5 ± 141.5	105–532 (*n* = 17) 355.5 ± 143.6
HL	20.57–67.94 (*n* = 63) 44,24 ± 33,49	21.65–78.61 (*n* = 66) 44.5 ± 15.6	30.04–55.48 (*n* = 22) 47.4 ± 7.3	32.63–69.21 (*n* = 17) 46.7 ± 10.3
HW	7.24–21.54 (*n* = 67) 10,0 ± 0,17	7.19–21.77 (*n* = 69) 13.9 ± 4.4	9.12–17.89 (*n* = 22) 14.9 ± 2.6	9.80–47.25 (*n* = 17) 15.5 ± 8.5
DN	2.89–12.93 (*n* = 68) 10,24 ± 0,71	3.51–11.97 (*n* = 69) 7.6 ± 4.9	4.17–9.47 (*n* = 22) 7.5 ± 1.4	4.78–10.68 (*n* = 17) 7.0 ± 1.6
EN	3.19–12.55 (*n* = 68) 9,98 ± 0,18	3.70–14.01 (*n* = 69) 7.3 ± 2.5	5.09–9.44 (*n* = 22) 7.8 ± 1.2	4.88–11.58 (*n* = 17) 7.3 ± 1.8
ED	4.19–8.55 (*n* = 68) 6,37 ± 3,08	4.12–8.41 (*n* = 69) 6.1 ± 1.3	4.85–7.64 (*n* = 22) 6.4 ± 0.8	5.08–7.95 (*n* = 17) 6.2 ± 0.8
AC	7.17–25.63 (*n* = 61) 10,21 ± 0,33	8.03–26.76 (*n* = 62) 16.3 ± 6.0	11.39–21.26 (*n* = 22) 16.8 ± 2.8	9.98–27.64 (*n* = 17) 15.9 ± 4.4

**Notes.**

In parenthesis the sampled number (*n*).

SVL snout-vent length (from the tip of the snout to the cloaca) TL tail length HL head length (from the tip of the snout to the quadratemandibular articulation) HW head width (length of the widest part of head) DN distance between nostrils (maximum distance between the nostrils) EN distance between eye and nostril ED eye diameter HH head height (maximum distance between the base of the mandible and the parietal surface

### Hemipenial morphology

We prepared a hemipenis from a topotype of *Hydrodynastes melanogigas* and 19 from *H. gigas* from different localities in the Amazon, East Brazil and La Plata hydrobasins (Appendix S1). Whenever possible, we prepared the hemipenes on the right side according to the technique originally described by Manzani & Abe (1988), as modified by Pesantes (1994), Zaher (1999), and Zaher & Prudente (2003). We stained the external calcareous structures with alizarin red, as suggested by Nunes et al. (2012), for a better visualization of microstructures in the surface of the organ. Terminology follows Dowling & Savage (1960), Zaher (1999) and Myers & Cadle (2003).

## Results

### Molecular approach

**Figure 2:**
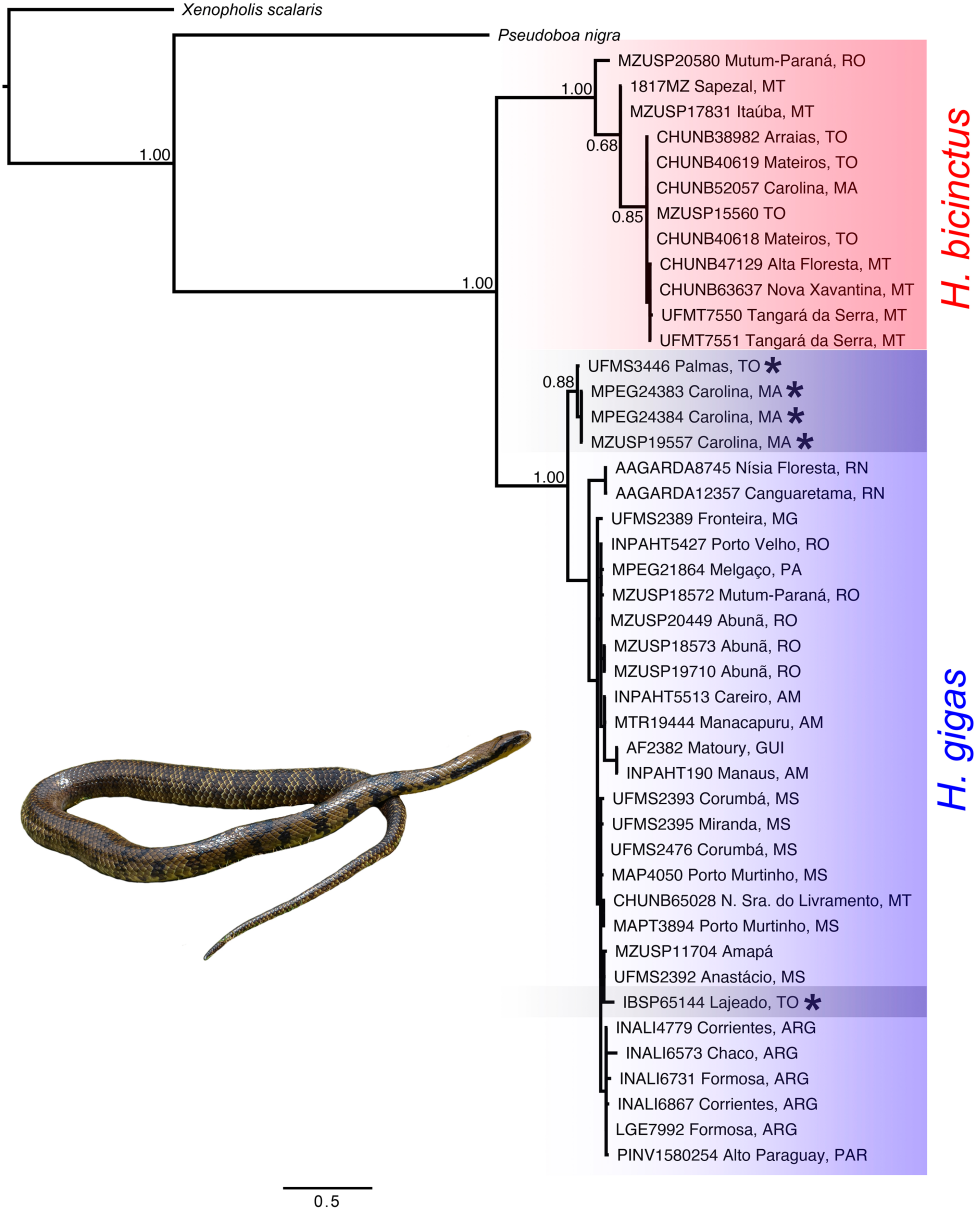
Concatenated tree (16S, Cytb, C-mos and NT3) of the genus *Hydrodynastes* recovered by Bayesian analysis in MrBayes. Numbers near the nodes correspond to support values indicate by posterior probability (pp). Asterisks indicate samples identified as *H. melanogigas*. Photo credit: Karoline Ceron.

We recovered the genus *Hydrodynastes* as monophyletic, and the topology of the concatenated gene tree showed two strongly supported clades with posterior probability ( *pp* = 1.00). Our concatenated dataset tree grouped *Hydrodynastes melanogigas* within *H*. *gigas* (Fig. 2). The uncorrected p-distance for both the mtDNA 16S and Cytb showed low genetic differences (0.01% and 0.2%, respectively) between the lineages of *H*. *gigas* and *H*. *melanogigas.* However, the genetic differences between *H*. *gigas* and *H*. *bicinctus* were 0.43% for 16S and 13% for Cytb (Table 5). Intraspecific variation in *H*. *gigas* was 0.0% to 0.04% for 16S and 0.0% to 0.17% for Cytb, while in *H. bicinctus* it was 0.0% for 16S and 0.0% to 0.23% for Cytb (Supplementary Material).

### Morphological approach

**Figure 3:**
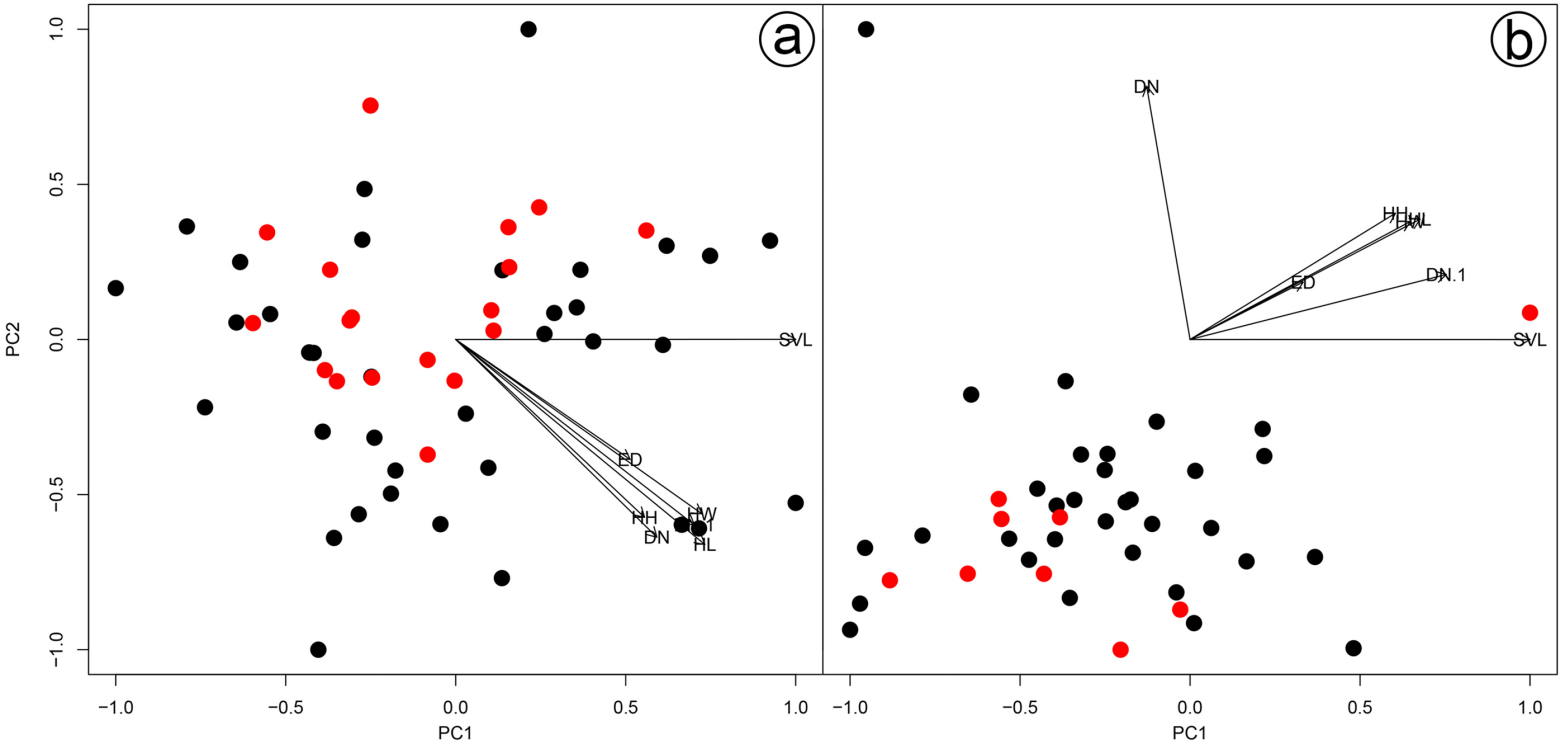
Results of a Principal Component Analysis (PCA) on the morphometric variables. Males (A) and females (B) of *Hydrodynastes gigas* and *H. melanogigas*. Black circle corresponds to *H. gigas* and red circle to *H. melanogigas*. SVL, snout-vent length; HL, head length; HW, head width; DN, distance between nostrils; EN, distance between eye and nostril; ED, eye diameter; HH, head height.

**Figure 4:**
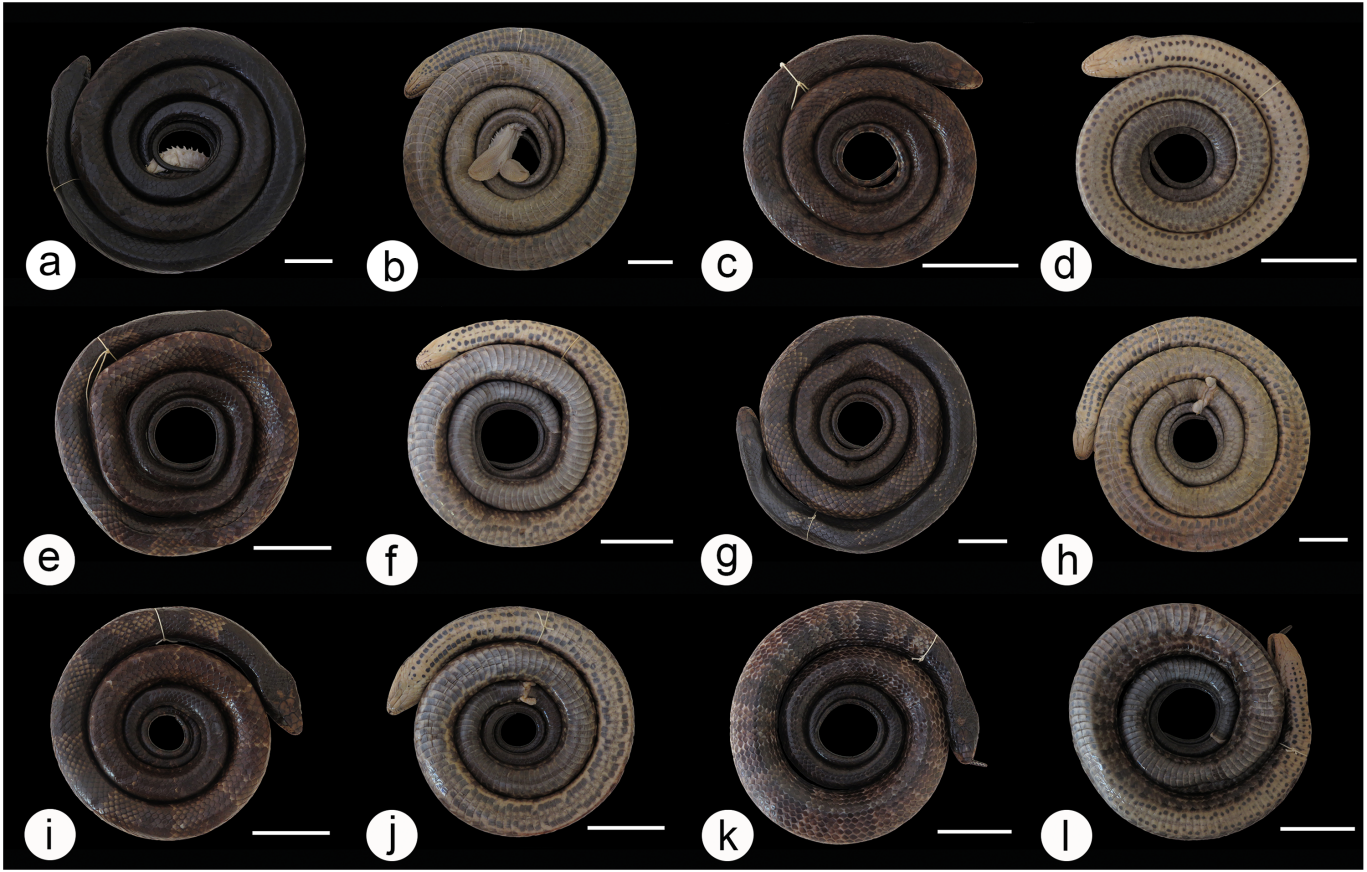
Dorsal and ventral view of the melanism gradient in *Hydrodynastes melanogigas*. MZCEULP 1218 (A, B); MZCEULP 516 (C D,) ; MZCEULP 1046 (E, F); MZCEULP 1273 (G, H); MZCEULP 758 (I, J); MZCEULP 938 (K, L). All specimens from Tocantins-Araguaia basin. Scale of 50 mm.

**Figure 5:**
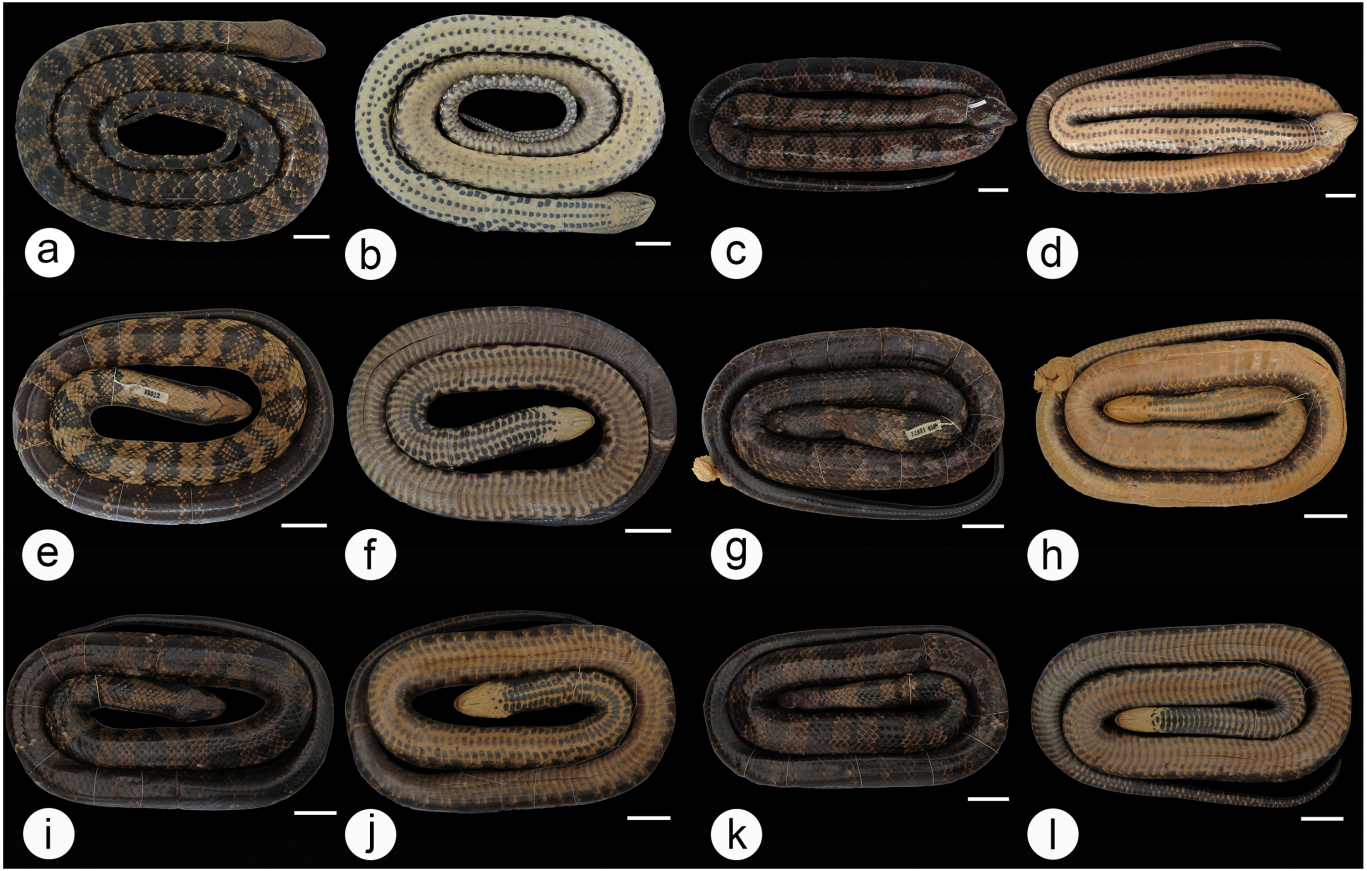
Dorsal and ventral view of the coloration gradient in *Hydrodynastes gigas*. La Plata basin UFMT 9076 (A, B); Amazon basin CHUNB 15159 (C, D); Tocantins-Araguaia basin MPEG 18012 (E, F), MPEG 18071 (G, H), MPEG 18046 (I, J), MPEG 18070 (K, L). Scale of 50 mm.

We found overlap in all meristic and morphometric characteristics between *Hydrodynastes gigas* and *H*. *melanogigas* (Tables 3 and 4). The first principal components from both PCA analysis (males and females) recovered 99% of variation, and through the MANOVA of males (*F* = 2.2949; *p* = 0.1109) and females (*F* = 0.3463; *p* = 0.7095) we found no significant morphometric differences between both species (Fig. 3). In addition, we observed gradient levels of melanism in *H. melanogigas* (Figs. 4A–4L). We examined fully melanic specimens (Fig. 4A) to specimens with clear visible bands along the body (Fig. 4K). We also observed that some *H*. *gigas* individuals from the Amazon, La Plata and Tocantins-Araguaia basins present darker coloration overlapping the gradient of melanism found in *H. melanogigas* (Figs. 5A–5L). We did not find any morphological characteristics that differentiate the two species.

**Table 5:** Unconrrected *p-distance* of 16S (lower left) and Cytb (upper right) mitochondrial fragment gene for the genus *Hydrodynastes*. Bold the *p-distance* between *H. gigas* and *H. melanogigas* for the two genes.

		1	2	3	4	5
1	*Xenopholis scalaris*		0.367	0.347	0.376	0.366
2	*Pseudoboa nigra*	0.084		0.366	0.313	0.341
3	*Hydrodynastes bicinctus*	0.126	0.118		0.129	0.123
4	*Hydrodynastes gigas*	0.128	0.097	0.043		**0.020**
5	*Hydrodynastes melanogigas*	0.130	0.099	0.042	**0.011**	

**Figure 6:**
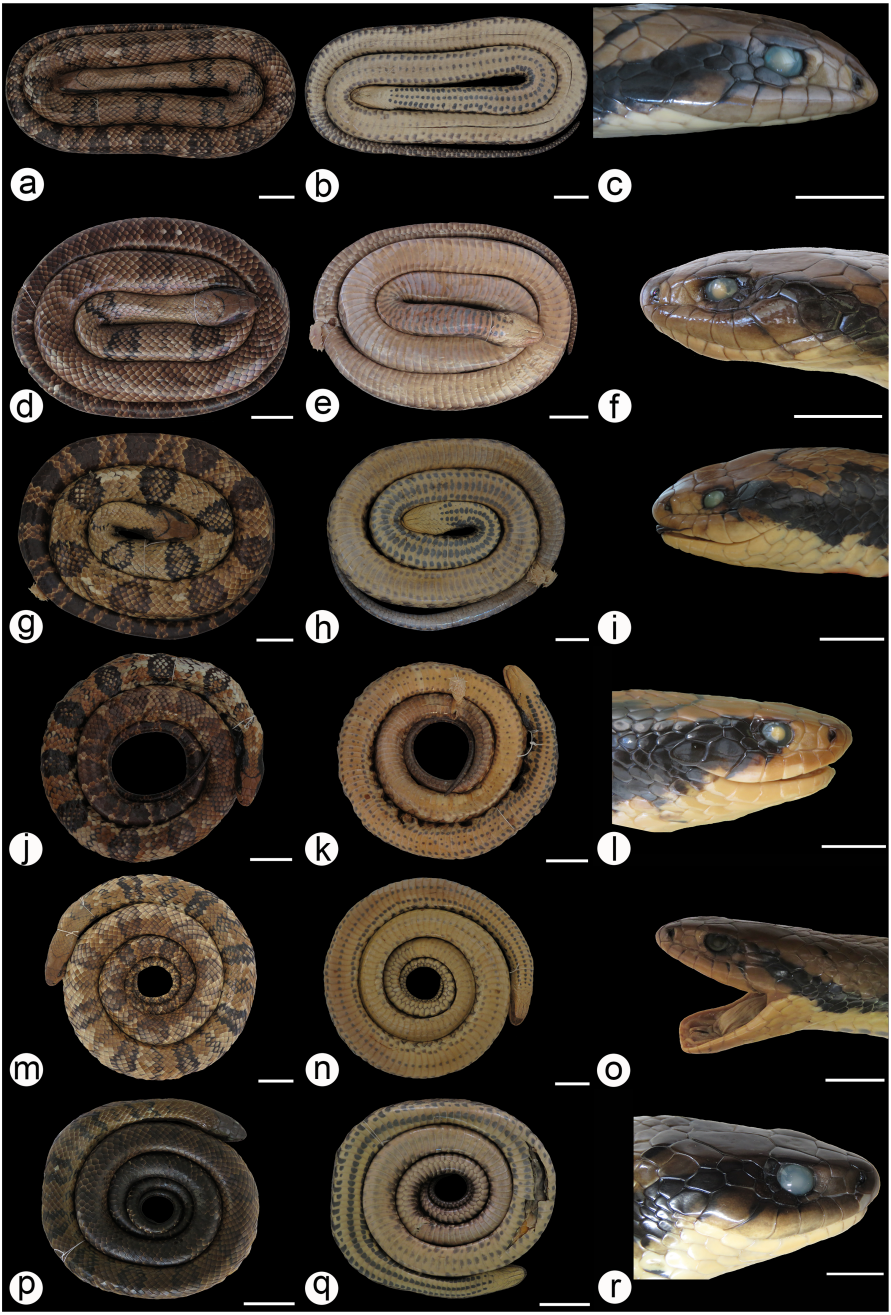
Dorsal and ventral view of the body and lateral view of the head in *Hydrodynastes gigas*. Amazon basin MPEG 22652 (A, B, C), CHUNB 56729 (D, E, F), MPEG 18674 (G, H, I); East Brazil CHUFPB 4611 (J, K, L); La Plata Basin UFMT 026 (M, N, O), ZUFMS 2389 (P, Q, R). Scale of 50 mm for body view and scale of 20 mm head view.

**Figure 7:**
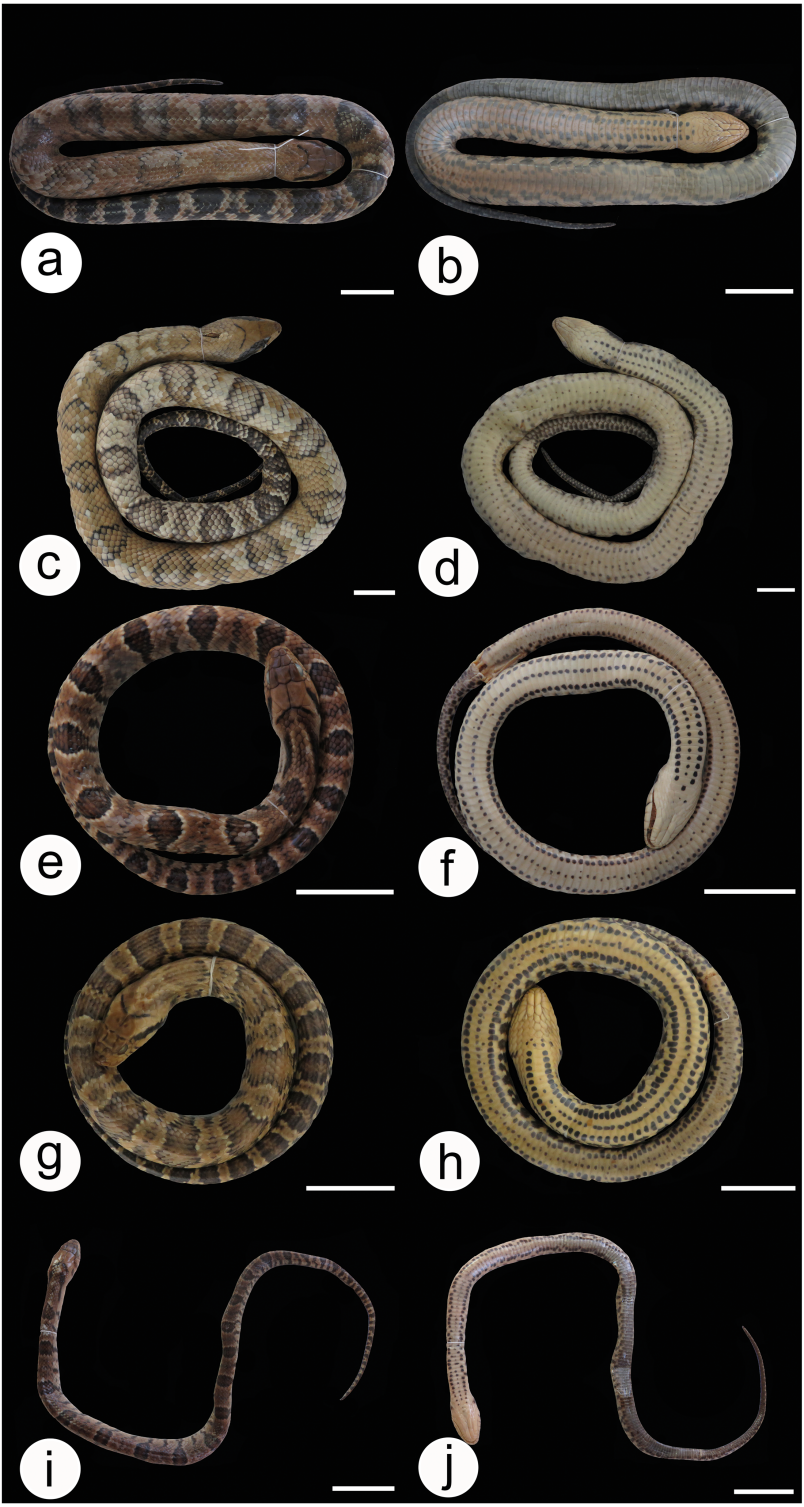
Dorsal and ventral view of the ontogenetic variation in *Hydrodynastes gigas*. Amazon basin CHUNB 66534 (A, B); Parnaiba basin CZDP 077 (C, D); East Brazil CHUFPB 14837 (E, F); La Plata basin ZUFMS 1603 (G, H); Tocantins-Araguaia basin CHUNB 22053 (I, J). Scale of 30 mm.

We did not observe coloration patterns within or between the populations of *Hydrodynastes gigas* (Figs. 6A–6R). We observed ontogenetic variation in the color pattern of all populations analyzed, with no distinction between males and females. Juveniles in the early stages of life have well-defined rounded dark spots all over their backs to the end of their tails; these spots are outlined by a lighter line, while in adults rounded spots may or may not be well defined, and may not present a clear outline (Figs. 7A–7J). Furthermore, we identified two young males of *H. gigas* (CHUNB 22053, Figs. 7I–7J); CHUNB 22068) from the type locality of *H*. *melanogigas,* as well as 18 more specimens from the Tocantins-Araguaia Basin. We provide more details in the “variation” section below.

**Figure 8:**
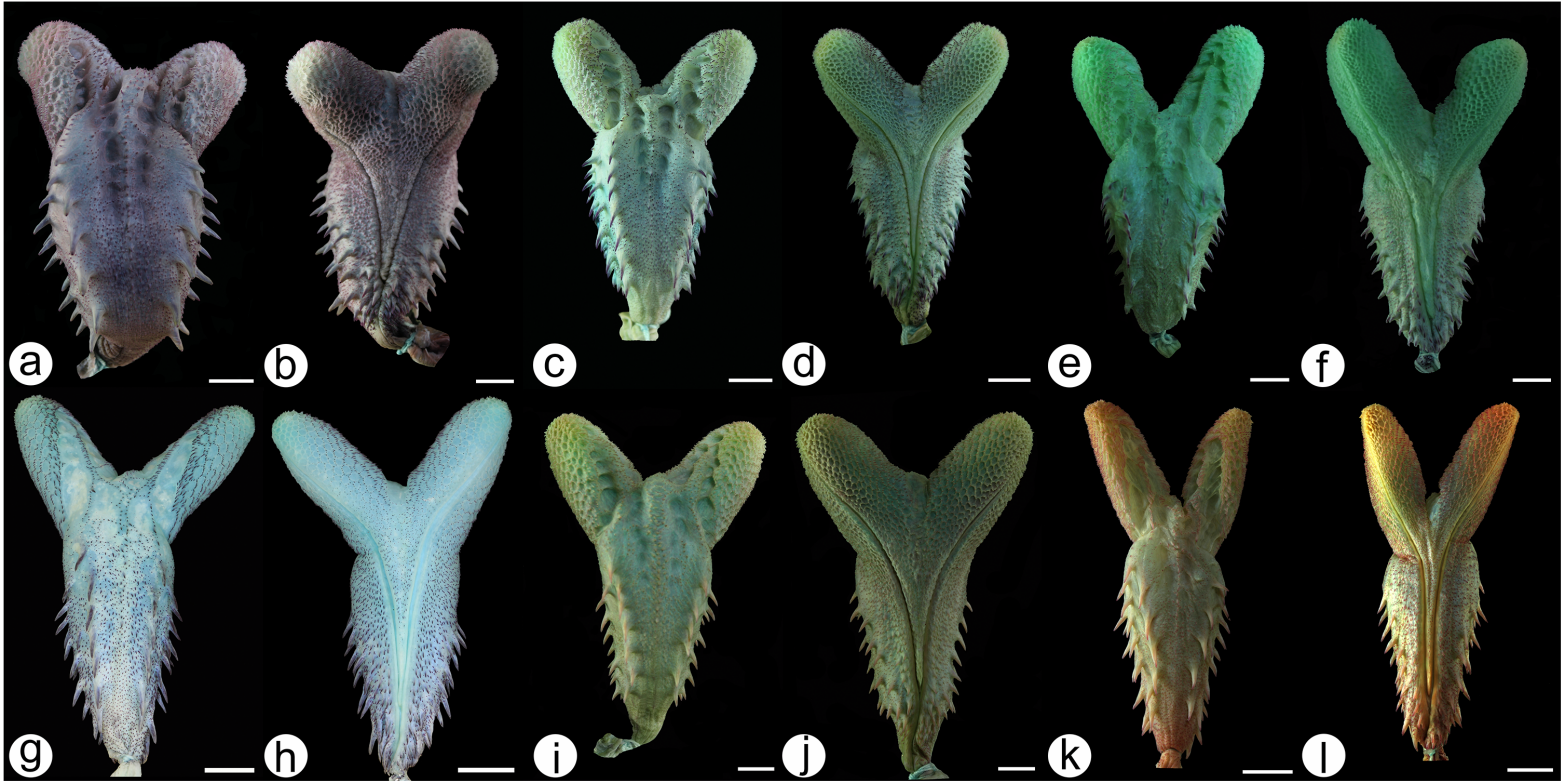
Hemipenial morphology. *Hydrodynastes gigas*: Amazon basin MZUSP 18572 (A) asulcate and (B) sulcate side. Northeast South America basin MPEG 25438 (C) asulcate and (D) sulcate side. La plata basin ZUFMS 1910 (E) asulcate and (F) sulcate side; MHNCI 4511 (G) asulcate and (H) sulcate side; UFSM 1937 (I) asulcate and (J) sulcate side. *Hydrodynastes melanogigas* Tocantins-Araguaia basin CHUNB 12802 (K) asulcate and (L) sulcate side. Scale of 10 mm.

***Hemipenis morphology*** (Figs. 8A–8L): When fully everted and expanded, hemipenes of *H. melanogigas* and *H*. *gigas* are undistinguishable (Figs. 8K–8L). The hemipenis is deeply bilobed, semicaliculate and semicapitate, with three or four vertical rows of large spines arranged on each side of the body. The body of the hemipenis is covered by spikes on the sulcate and assulcate faces. The sulcus spermaticus bifurcates in the proximal region of the hemipenis body and each branch extends centrolinearly until it reaches the proximal region of the lobes, in which they follow a centrifugal position that ends at the lateral region of the tip of each. The capitulum, formed by papillate calyces and spikes, extends over most of the surface of the lobes, except in the region of the assulcate face that is occupied by two parallel rows of papillated and conspicuous body calyces that extend to the distal region of the hemipenis body, where they converge on the lobular crest and continues to the middle portion of the hemipenis. We detected low intraspecific variation among *Hydrodynastes gigas* populations. Some hemipenes showed little size variation in lobes and body calyces, varying from slightly visible to conspicuous in the hemipenial body.

## Discussion

*Hydrodynastes gigas* is widely distributed throughout South America, occurring with low genetic variability throughout most of its extension range. Although the genetic structure of widely distributed species can be easily influenced by natural barriers (Patton, Da Silva & Malcolm, 1994; Pellegrino et al., 2005; Rocha et al., 2015), this clearly is not the case for the genus *Hydrodynastes* (see Murta-Fonseca, Franco & Fernandes, 2015).

Here, we used an integrative taxonomic approach and adopted the species concept of one lineage with distinct evolutionary histories (De Queiroz, 2007), to test the taxonomic validity of *Hydrodynastes melanogigas*. Our results did not separate *H*. *gigas* and *H*. *melanogigas* based on molecular, meristic, morphometric and hemipenial characters. The hemipenis of *Hydrodynastes melanogigas* analyzed showed no differences from the hemipenis of *H. gigas* (*n* = 19). In their description of *H. melanogigas*, Franco, Fernandes & Bentim (2007) also pointed out its similarity with *H. gigas* based on meristic characters and hemipenis morphology. The only superficial distinction that remains between these two taxa is the presence of melanism in the latter. Geographic or regional melanism has already been reported for several populations of Squamata (Pearse & Pogson, 2000; King, 2003; Bernardo et al., 2012). In addition, polychromatism can be a bias within taxonomy, if the revision and/or description of species does not take into account the organisms throughout their whole distribution (Bernardo et al., 2012; Ruane et al., 2018; Mângia et al., 2020). In fact, Franco, Fernandes & Bentim (2007) carried out an analysis covering almost the entire distribution of *H. gigas*, however we found degrees of melanism in some populations that were not identified by other authors. The variation of melanism found in *H. gigas* and the melanistic gradient observed in *H. melanogigas* (Figs. 4 and 5), along with genetic support, suggests that *H. melanogigas* is not a distinct species but rather a melanic population of *H*. *gigas*. The distribution of *Hydrodynastes melanogigas* without sympatry with *H. gigas* in the Tocantins-Araguaia basin was an important factor for its description (Franco, Fernandes & Bentim, 2007). However, in this study, we analyzed two juveniles of *H*. *gigas* from the type locality of *H*. *melanogigas* (CHUNB 22053, Figs. 7I–Figs. 7J; CHUNB 22068). All specimens analyzed by Franco, Fernandes & Bentim (2007) and herein were adults or juveniles and no neonates were observed. Therefore, we do not know whether the specimens considered as *H. melanogigas* could have been born melanic or if melanism occurs during their ontogeny. Still, some studies suggest that thermal melanism is associated with latitude and high altitudes, i.e., relatively cold environments (Capula, Luiselli & Monney, 1995), which does not agree with the present case. More studies are needed to confirm the adaptive meaning of melanism through studies of thermal biology. Therefore, due to the lack of any characteristic that sustain these two taxa as distinct species and their low genetic distance (0.04% 16S and 0.2% Cytb), we consider *H. melanogigas* Franco, Fernandez & Bentim, 2007 as a junior synonym of *H. gigas* (Duméril, Bibron & Duméril, 1854).

### Systematic account

**Table:** 

***Hydrodynastes gigas*** (Duméril, Bibron & Duméril, 1854)
*Xenodon gigas*Duméril, Bibron & Duméril, 1854. Erpétologie générale vol. 7: 761.
*Lejosophis gigas*Jan, 1863. Elenco Sistematico degli Ofidi descriti e disegnati per l’Iconografia Generale. vol. 2: 56.
*Cyclagras gigas*Cope, 1885. Proceeding of the American Philosophical Society: 185.
*Cyclagras gigas*Boulenger, 1894. Catalogue of the snakes in the British Museum vol. 2: 144.
*Lejosophis gigas*Dunn, 1944. Caldasia: 69.
*Lejosophis gigas*Hoge, 1958. Papéis Avulsos de Zoologia: 222.
*Hydrodynastes gigas*Hoge, 1966. Ciência e Cultura: 143.
*Cyclagras gigas*Peters & Orejas-Miranda, 1970. Catalogue of the Neotropical Squamata. Part I: 78.
*Hydrodynastes gigas*Dowling & Gibson, 1970. Herpetological Review (2): 38
*Hydrodynastes melanogigas*Franco, Fernandes & Bentim, 2007 Zootaxa (1613): 58. **New Synonymy**


Type material: syntype MNHN 3623

Type locality: Corrientes Province, Argentina.

**Figure 9:**
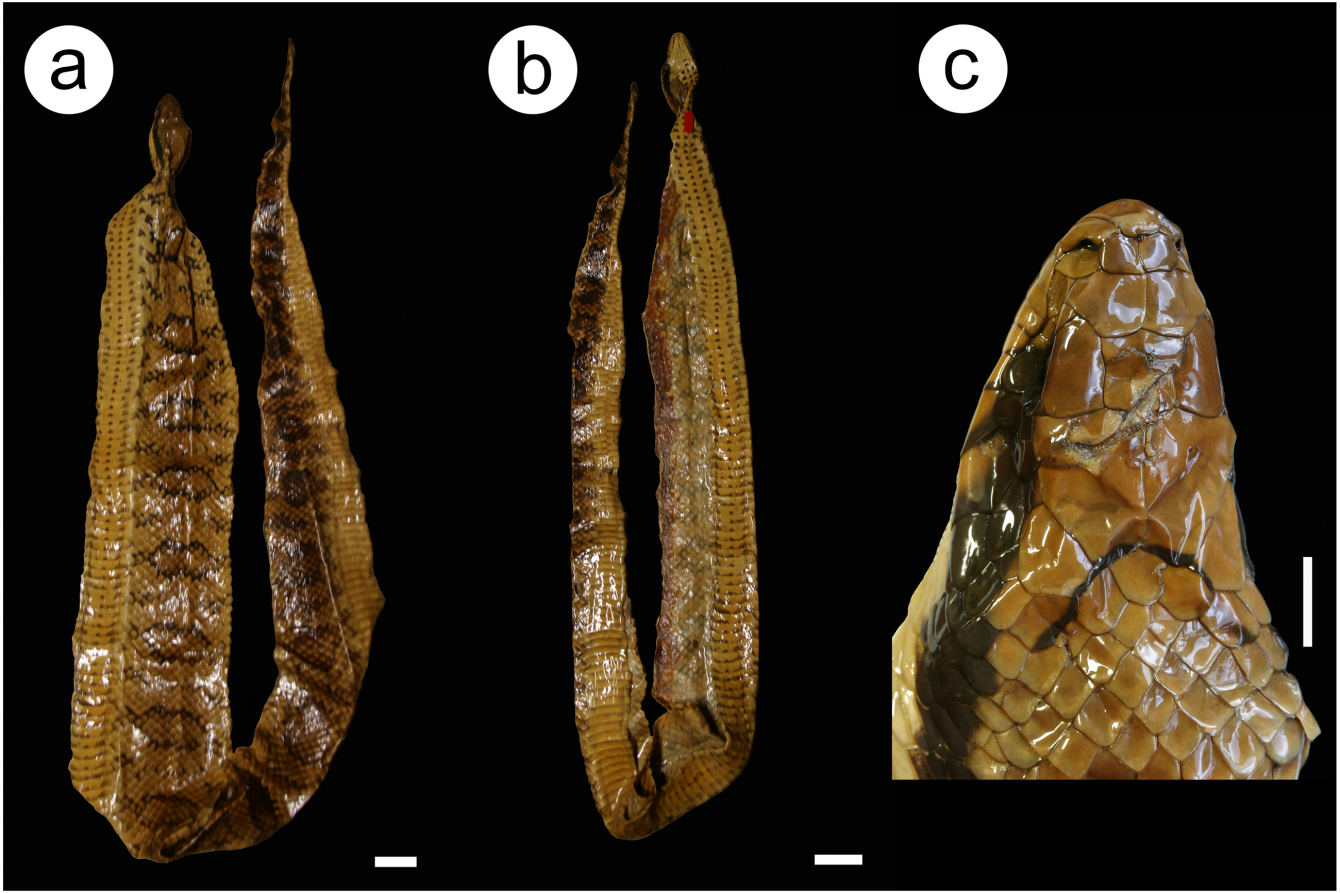
Lectotype MNHN 3623 Hydrodynastes gigas. Dorsal (A) and ventral (B) views of the body and head view (C). Credit: Hussam Zaher. Scale of 50 mm for body view and scale of 10 mm head view.

**Comments on the type series:** Duméril, Bibron and Duméril’s (1854) description of *Xenodon gigas* did not refer to any voucher specimen. The authors only cited that M. A. d’Orbigny collected three individuals in Rio de La Plata, Corrientes Province, Argentina (without further information). The authors also mentioned a plate (Xénodon géant. Atlas, pi. 76, fig-5), which only presents a cranial picture. Overall, Wallach, Williams & Boundy (2014, p. 339) indicate that the types would be MNHN 2493 a-c, but according to Uetz et al. (2019), it would be an individual labeled MNHN 3623. Due to this conflicting information, we contacted the curator of the Herpetological Collection at the Muséum National d’Histoire Naturelle de Paris (Dr. N. Vidal) who confirmed that there is only one type specimen, a skin labeled MNHN 3623 (Fig. 9), deposited in the collection, and that the other two specimens appear to be lost . Since Duméril, Bibron & Duméril (1854) did not select any specific specimen from their type series, we therefore, designate the specimen MNHN 3623 as the lectotype of *Hydrodynastes gigas*.

**Description of the lectotype MNHN 3623** (Fig. 9): Adult of undetermined sex; SVL 1570 mm; TL 540 mm; HL 62 mm; HW 35 mm; DN 10 mm; two internasals; nasal divided; one loreal; one preocular; three suboculars; two postoculars; temporal 2 + 2∕2 + 2 ; nine supralabials, none contacting the orbit; eleven infralabials, first to sixth contacting chin shields on the right side and first to fifth on the left side; two pairs of chin shields; dorsal 19/16/15 scales, smooth; two apical pits; ventral 153; subcaudals 74, paired and cloacal scale single.

**Color of the preserved lectotype (ethanol 70%)** (Fig. 9): Head brown with black ‘U’ shaped spot at the end of the parietal scale; post-ocular stripe that extends longitudinally on each side; supralabial brown with the last four scales stained black; infralabials and chin shields cream; dorsum of body brown with dark rounded spots that extend to the end of the tail; ventral body cream with three black stripes to the middle of the body.

**Diagnosis:** *Hydrodynastes gigas* is distinguished from its congener *H. bicinctus* by the following combination of characters: dorsal scales normally 19/19/15; ventral scales in males 150–168 and in females 152–172; subcaudal scales in males 58–88 and in females 49–84; maxillary teeth 15–17; two apical pits in the dorsal scales; post-ocular stripe that extends longitudinally (on each side); ventral body with three lines of continuous spots up to the middle of the body.

**Variation**: All variation in morphometric and meristic data are presented in Tables 3 and 4. Regarding coloration patterns, a considerable degree of color variation can be found in *H*. *gigas* (Figs. 4A–4L; 5A–5L; 6A–6R and 7A–7J) dorsum ranging from yellow to dark brown or completely dark in melanic populations; rounded spots on the dorsum may vary in shape and size, in some individuals they may be hollow or filled; in neonates the dark rounded spots are well defined throughout the dorsum until the end of the tail, and these spots are outlined by a lighter line; darks spot in the shape of ‘V’ or ‘U’ at the end of the parietal scale, only visible in non-melanic populations; ventral body cream/brown with three black stripes that usually go up to the middle of the body, rarely to the cloaca, some individuals do not have these stripes, in melanic populations the belly is dark without spots or when present these spots are continuous on the sides.

**Figure 10:**
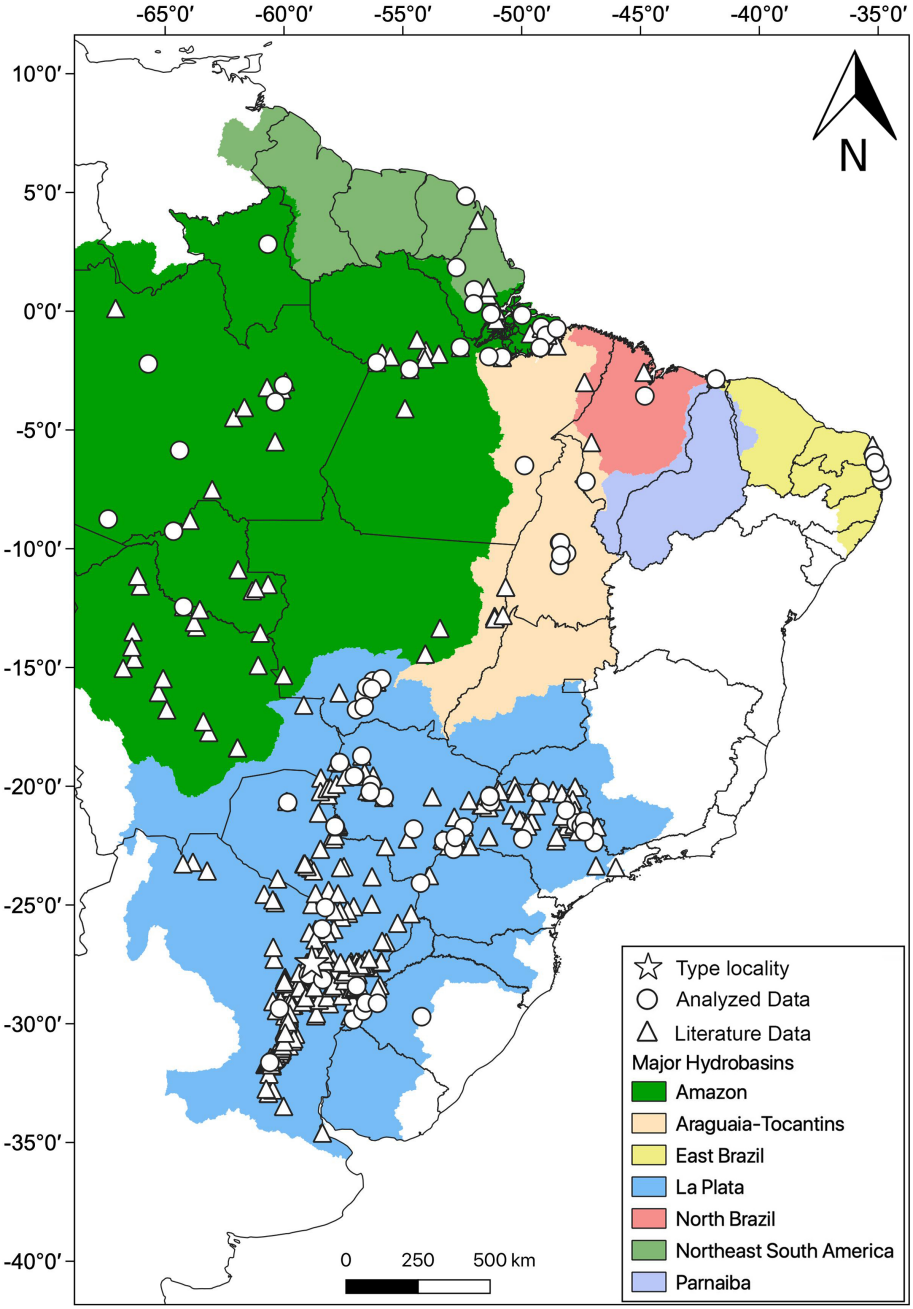
Geographic distribution of *Hydrodynastes gigas* in South America. We compiled data from the literature, specimens and tissues analyzed.

**Distribution**: *Hydrodynastes gigas* is widely distributed throughout South America, east of the Andes, occurring in Amazon, East Brazil, La Plata, North Brazil, Northeast South America, Parnaiba and Tocantins-Araguaia hydrobasins (Fig. 10).

## Conclusions

Our results did not separate *H*. *gigas* and *H*. *melanogigas* based on molecular, meristic, morphometric and hemipenial characters. Therefore, the melanistic pattern of *Hydrodynastes melanogigas* is characterized here as the result of polymorphism within *H. gigas*. Although our integrative approach helped elucidate the taxonomic status of *H. melanogigas*, we believe future, multi-loci phylogeographic studies are needed in order to better understand the evolutionary history of the populations belonging to the two remaining species *H*. *gigas* and *H*. *bicinctus*.

##  Supplemental Information

10.7717/peerj.10073/supp-1 Appendix S1 Specimens examined * prepared hemipenes.

10.7717/peerj.10073/supp-2 Supplemental Information 2 Uncorrected *p-distance* of 16S and Cytb mitochondrial fragment gene for all used samples of the genus *Hydrodynastes*

10.7717/peerj.10073/supp-3 Supplemental Information 3 16S mitochondrial fragment gene sequences of the samples used of the genus *Hydrodynastes.*

10.7717/peerj.10073/supp-4 Supplemental Information 4 Cmos nuclear fragment gene sequences of the samples used of the genus *Hydrodynastes.*

10.7717/peerj.10073/supp-5 Supplemental Information 5 Cytb mitochondrial fragment gene sequences of the samples used of the genus *Hydrodynastes.*

10.7717/peerj.10073/supp-6 Supplemental Information 6 NT3 nuclear fragment gene sequences of the samples used of the genus *Hydrodynastes*

10.7717/peerj.10073/supp-7 Supplemental Information 7 16S, Cytb, Cmos and NT3 fragment gene concatenated sequences of the samples used of the genus Hydrodynastes

10.7717/peerj.10073/supp-8 Supplemental Information 8 Raw Data

## Additional Information and Declarations

### DNA Deposition

The following information was supplied regarding the deposition of DNA sequences: The 16S sequences are available at Genbank: MT192267–MT192299; MT215327–MT215335. The Cytb sequences are available at Genbank: MT224972–MT225014. The Cmos sequences are available at Genbank: MT328065–MT328096. The NT3 sequences are available at Genbank: MT328097–MT328134 and MT424769. In addition, all information is available in Table 1.

### Data Availability

The following information was supplied regarding data availability: Summarized morphometric and meristic data are available in Tables 3 and 4. Location data and zoological collections are available in Appendix S1. The data shows overlap in all meristic and morphometric characteristics between *Hydrodynastes gigas* and *H. melanogigas*.

## References

[ref-1] Andrén C, Nilson G (1981). Reproductive success and risk of predation in normal and melanistic colour morphs of the adder, *Vipera berus*. Biological Journal of the Linnean Society.

[ref-2] Bernardo PH, Machado FA, Murphy RW, Zaher H (2012). Redescription and morphological variation of *Oxyrhopus clathratus* (Duméril, Bibron & Duméril, 1854) (Serpentes: Dipsadidae: Xenodontinae). South American Journal of Herpetology.

[ref-3] Bickford D, Lohman DJ, Sodhi NS, Ng PK, Meier R, Winker K, Ingram KK, Das I (2007). Cryptic species as a window on diversity and conservation. Trends in Ecology & Evolution.

[ref-4] Boulenger GA (1894). Catalogue of the snakes in the British Museum (Natural History), Vol. II.

[ref-5] Briolat ES, Burdfield-Steel ER, Paul SC, Rönkä KH, Seymoure BM, Stankowich T, Stuckert AM (2019). Diversity in warning coloration: selective paradox or the norm?. Biological Reviews.

[ref-6] Brusquetti F, Jansen M, Barrio-Amorós C, Segalla M, Haddad CF (2014). Taxonomic review of *Scinax fuscomarginatus* (Lutz, 1925) and related species (Anura; Hylidae). Zoological Journal of the Linnean Society.

[ref-7] Capula M, Luiselli L, Monney JC (1995). Correlates of melanism in a population of adders (*Vipera berus*) from the Swiss Alps and comparisons with other alpine populations. Amphibia-Reptilia.

[ref-8] Cope ED (1885). Twelfth contribution to the herpetology of tropical America. Proceedings of the American Philosophical Society.

[ref-9] Costa HC, Moura MR, Feio RN (2013). Taxonomic revision of *Drymoluber* Amaral, 1930 (Serpentes: Colubridae). Zootaxa.

[ref-10] Dayrat B (2005). Towards integrative taxonomy. Biological Journal of the Linnean society.

[ref-11] De Queiroz K (2007). Species concepts and species delimitation. Systematic Biology.

[ref-12] Dowling HG (1951). A proposed standard system of counting ventrals in snakes. British Journal of Herpetology.

[ref-14] Dowling HG, Gibson FW (1970). Relationship of the Neotropical snakes *Hydrodynastes bicinctus* and *Cyclagras gigas*. Herpetological Review.

[ref-15] Dowling HG, Savage JM (1960). A guide to the snake hemipenis: a survey of basic structure and systematic characteristics. Zoologica.

[ref-16] Duméril AMC, Bibron G, Duméril A (1854). Erpétologie Genérale ou Histoire Naturelle Complete des Reptiles. Vol. VII.

[ref-17] Dunn ER (1944). *Dugandia*, a new snake genus for the *Coluber bicinctus* Hermann. Caldasia.

[ref-18] Edgar RC (2004). MUSCLE: multiple sequence alignment with high accuracy and high throughput. Nucleic Acids Research.

[ref-19] Entiauspe-Neto OM, De Sena A, Tiutenko A, Loebmann D (2019). Taxonomic status of *Apostolepis barrioi* Lema, 1978, with comments on the taxonomic instability of *Apostolepis* Cope, 1862 (Serpentes, Dipsadidae). ZooKeys.

[ref-20] Forsman A, Ås S (1987). Maintenance of colour polymorphism in adder, *Vipera berus*, populations: a test of a popular hypothesis. Oikos.

[ref-21] França DP, Barbo FE, Silva-Junior NJ, Silva HL, Zaher H (2018). A new species of *Apostolepis* (Serpentes, Dipsadidae, Elapomorphini) from the Cerrado of Central Brazil. Zootaxa.

[ref-22] Franco FL, Fernandes DS, Bentim BM (2007). A new species of *Hydrodynastes* Fitzinger, 1843 from central Brazil (Serpentes: Colubridae: Xenodontinae). Zootaxa.

[ref-23] Franco FL, Trevine VC, Montingelli GG, Zaher H (2017). A new species of *Thamnodynastes* from the open areas of central and northeastern Brazil (Serpentes: Dipsadidae: Tachymenini). Salamandra.

[ref-24] Giraudo AR, Scrocchi GJ (2002). Argentinian snakes: an annotated checklist. Smithsonian Herpetological Information Service.

[ref-77] Grazziotin FG, Zaher H, Murphy RW, Scrocchi G, Benavides MA, Zhang YP, Bonatto SL (2012). Molecular phylogeny of the new world Dipsadidae (Serpentes: Colubroidea): a reappraisal. Cladistics.

[ref-25] Henderson RW (1997). A taxonomic review of the *Corallus hortulanus* complex of Neotropical tree boas. Caribbean Journal of Science.

[ref-26] Henderson RW, Passos P, Feitosa D (2009). Geographic variation in the emerald treeboa, *Corallus caninus* (Squamata: Boidae). Copeia.

[ref-27] Hoge AR (1958). Três notas sobre serpentes brasileiras. Papéis Avulsos do Departamento de Zoologia. Secretaria da Agricultura–São Paulo–Brasil.

[ref-28] Hoge AR (1966). Notes on *Hydrodynastes* [Serpentes—Colubridae]. Ciência e Cultura.

[ref-29] Jan G (1863). Elenco sistematico degli Ofidi descritti e disegnati per l’Iconographia Generale.

[ref-30] Kettlewell HBD (1973). The evolution of melanism.

[ref-31] King RB (2003). Mendelian inheritance of melanism in the garter snake *Thamnophis sirtalis*. Herpetologica.

[ref-32] Lanfear R, Frandsen PB, Wright AM, Senfeld T, Calcott B (2016). PartitionFinder 2: new methods for selecting partitioned models of evolution for molecular and morphological phylogenetic analyses. Molecular Biology and Evolution.

[ref-33] Lawson R, Slowinski JB, Crother BI, Burbrink FT (2005). Phylogeny of the Colubroidea (Serpentes): new evidence from mitochondrial and nuclear genes. Molecular Phylogenetics and Evolution.

[ref-34] Mângia S, Oliveira EF, Santana DJ, Koroiva R, Paiva F, Garda AA (2020). Revising the taxonomy of *Proceratophry* s Miranda- Ribeiro, 1920 (Anura: Odontophrynidae) from the Brazilian semiarid Caatinga: Morphology, calls and molecules support a single widespread species. Journal of Zoological Systematics and Evolutionary Research.

[ref-35] Manzani PR, Abe AS (1988). Sobre dois novos métodos de preparo do hemipenis de serpentes. Memórias do Instituto Butantan.

[ref-36] Meneses-Pelayo E, Passos P (2019). New polychromatic species of *Atractus* (Serpentes: Dipsadidae) from the eastern portion of the Colombian Andes. Copeia.

[ref-37] Moraes-Da-Silva A, Amaro RC, Nunes PMS, Strüssmann C, Teixeira MJ, Andrade AJ, Sudré V, Recoder R, Rodrigues MT, Curcio FF (2019). Chance, luck and a fortunate finding: a new species of watersnake of the genus *Helicops* Wagler, 1828 (Serpentes: Xenodontinae), from the Brazilian Pantanal wetlands. Zootaxa.

[ref-38] Murta-Fonseca RA, Franco FL, Fernandes DS (2015). Taxonomic status and morphological variation of *Hydrodynastes bicinctus* (Hermann, 1804) (Serpentes: Dipsadidae). Zootaxa.

[ref-39] Myers CW, Cadle JE (2003). On the snake hemipenis, with notes on *Psomophis* and techniques of eversion: a response to Dowling. Herpetological Review.

[ref-40] Nogueira CC, Argôlo AJS, Arzaendia V, Azevedo JA, Barbo FE, Bérnils RS, Bolochio BE, Borges-Martins M, Brasil-Godinho M, Braz H, Buononato MA, Cisneros-Heredia DF, Colli GR, Costa HC, Franco FL, Giraudo A, Gonzalez RC, Guedes T, Hoogmoed MS, Marques OAV, Montingelli GG, Passos P, Prudente ALC, Rivas GA, Sanchez PM, Serrano FC, Silva Jr NJ, Strüssmann C, Vieira-Alencar JPS, Zaher H, Sawaya RJ, Martins M (2019). Atlas of Brazilian snakes: verified point-locality maps to mitigate the Wallacean shortfall in a megadiverse snake fauna. South American Journal of Herpetology.

[ref-41] Nunes PM, Fouquet A, Curcio FF, Kok PJ, Rodrigues MT (2012). Cryptic species in *Iphisa elegans* Gray, 1851 (Squamata: Gymnophthalmidae) revealed by hemipenial morphology and molecular data. Zoological Journal of the Linnean Society.

[ref-42] Oksanen J, Kindt R, Legendre P, O’Hara B, Stevens MHH (2007).

[ref-43] Padial JM, De La Riva I (2010). A response to recent proposals for integrative taxonomy. Biological Journal of the Linnean Society.

[ref-44] Padial JM, Miralles A, De la Riva I, Vences M (2010). The integrative future of taxonomy. Frontiers in Zoology.

[ref-45] Palumbi S, Martin A, Romano S, McMillan WO, Stice L, Grabowski G (2002).

[ref-46] Pante E, Schoelinck C, Puillandre N (2014). From integrative taxonomy to species description: one step beyond. Systematic Biology.

[ref-47] Passos P, Martins A, Pinto-Coelho D (2016). Population morphological variation and natural history of *Atractus potschi* (Serpentes: Dipsadidae) in Northeastern Brazil. South American Journal of Herpetology.

[ref-48] Passos P, Prudente ALC (2012). Morphological variation, polymorphism, and taxonomy of the *Atractus torquatus* complex (Serpentes: Dipsadidae). Zootaxa.

[ref-49] Patton JL, Da Silva MNF, Malcolm JR (1994). Gene genealogy and differentiation among arboreal spiny rats (Rodentia: Echimyidae) of the Amazon basin: a test of the riverine barrier hypothesis. Evolution.

[ref-50] Pearse DE, Pogson GH (2000). Parallel evolution of the melanic form of the California legless lizard, *Anniella pulchra*, inferred from mitochondrial DNA sequence variation. Evolution.

[ref-51] Pellegrino K, Rodrigues MT, Waite AN, Morando M, Yassuda YY, Sites JW (2005). Phylogeography and species limits in the *Gymnodactylus darwinii* complex (Gekkonidae, Squamata): genetic structure coincides with river systems in the Brazilian Atlantic Forest. Biological Journal of the Linnean Society.

[ref-52] Pereira-Filho GA, Montingelli GG (2006). Geographic distribution *Hydrodynastes gigas*. Herpetological Review.

[ref-53] Pesantes OS (1994). A method for preparing the hemipenis of preserved snakes. Journal of Herpetology.

[ref-54] Peters JA (1964). Dictionary of Herpetology—a brief and meaningful definition of words and terms used in herpetology.

[ref-55] Peters JA, Orejas-Miranda B (1970). Catalogue of the neotropical squamata. Part I. Snakes.

[ref-56] Pook CE, Wüster W, Thorpe RS (2000). Historical biogeography of the western rattlesnake (Serpentes: Viperidae: *Crotalus viridis*), inferred from mitochondrial DNA sequence information. Molecular Phylogenetics and Evolution.

[ref-76] Pyron RA, Burbrink FT, Colli GR, De Oca ANM, Vitt LJ, Kuczynski CA, Wiens JJ (2011). The phylogeny of advanced snakes (Colubroidea), with discovery of a new subfamily and comparison of support methods for likelihood trees. Molecular phylogenetics and evolution.

[ref-57] R Core Team (2014). https://www.R-project.org/.

[ref-58] Rambaut A, Suchard MA, Xie D, Drummond AJ (2014). http://beast.bio.ed.ac.uk/Tracer.

[ref-59] Recoder RS, De Pinho Werneck F, Teixeira Jr M, Colli GR, Sites Jr JW, Rodrigues MT (2014). Geographic variation and systematic review of the lizard genus *Vanzosaura* (Squamata, Gymnophthalmidae), with the description of a new species. Zoological Journal of the Linnean Society.

[ref-60] Rocha RG, Ferreira E, Loss AC, Heller R, Fonseca C, Costa LP (2015). The Araguaia river as an important biogeographical divide for didelphid marsupials in central Brazil. Journal of Heredity.

[ref-61] Ronquist F, Huelsenbeck JP (2003). MrBayes 3: Bayesian phylogenetic inference under mixed models. Bioinformatics.

[ref-62] Ruane S, Myers EA, Lo K, Yuen S, Welt RS, Juman M, Futterman I, Nussbaum RA, Schneider G, Burbrink FT, Raxworthy CJ (2018). Unrecognized species diversity and new insights into colour pattern polymorphism within the widespread Malagasy snake *Mimophis* (Serpentes: Lamprophiidae). Systematics and Biodiversity.

[ref-63] Sabaj-Pérez MH (2014). http://www.asih.org.

[ref-64] Sambrook J, Fritsch EF, Maniatis R (1989). Molecular cloning: a laboratory manual.

[ref-65] Santos Jr AP, Adams GB, Buhler D, Ribeiro S, Carvalho TS (2017). Distribution extension for *Hydrodynastes melanogigas* (Franco, Fernandes & Bentim, 2007) (Serpentes: Dipsadidae: Xenodontinae) in the Araguaia-Tocantins basin, Brazilian Cerrado. Check List.

[ref-66] Silva Jr NJ, Hamdan B, Tonial I, Silva HLR, Cintra C (2012). *Hydrodynastes melanogigas* (Franco, Fernandes & Bentim, 2007) (Squamata: Serpentes: Colubridae): range extension and new state record. Check List.

[ref-67] Tamura K, Stecher G, Peterson D, Filipski A, Kumar S (2013). MEGA6: molecular evolutionary genetics analysis version 6.0. Molecular Biology and Evolution.

[ref-68] Townsend TM, Alegre RE, Kelley ST, Wiens JJ, Reeder TW (2008). Rapid development of multiple nuclear loci for phylogenetic analysis using genomic resources: an example from squamate reptiles. Molecular Phylogenetics and Evolution.

[ref-69] Uetz P, Cherikh S, Shea G, Ineich I, Campbell PD, Doronin IV, Rosado J, Wynn A, Tighe KA, Mcdiarmid R, Lee JL, Köhler G, Ellis R, Doughty P, Raxworthy CJ, Scheinberg L, Resetar A, Sabaj M, Schneider G, Franzen M, Glaw F, Böhme W, Schweiger S, Gemel R, Couper P, Amey A, Dondorp E, Ofer G, Meiri S, Wallach V (2019). A global catalog of primary reptile type specimens. Zootaxa.

[ref-70] Vanzolini PE, Ramos-Costa AMM, Vitt LJ (1980). Répteis das caatingas.

[ref-71] Vidal N, Dewynter M, Gower DJ (2010). Dissecting the major American snake radiation: a molecular phylogeny of the Dipsadidae Bonaparte (Serpentes, Caenophidia). Comptes Rendus Biologies.

[ref-72] Wallach V, Williams KL, Boundy J (2014). Snakes of world: a catalogue of living and extinct species.

[ref-73] Zaher H (1999). Hemipenial morphology of the South American xenodontine snakes, with a proposal for a monophyletic Xenodontinae and a reappraisal of colubroid hemipenes. Bulletin of the American Museum of Natural History.

[ref-78] Zaher H, Grazziotin FG, Cadle JE, Murphy RW, Moura-Leite JCD, Bonatto SL (2009). Molecular phylogeny of advanced snakes (Serpentes, Caenophidia) with an emphasis on South American Xenodontines: a revised classification and descriptions of new taxa. Papéis Avulsos de Zoologia.

[ref-74] Zaher H, Murphy RW, Arredondo JC, Graboski R, Machado-Filho PR, Mahlow K, Montingelli GG, Quadros AB, Orlov NL, Wilkinson M, Zhang YP, Grazziotin FG (2019). Large-scale molecular phylogeny, morphology, divergence-time estimation, and the fossil record of advanced caenophidian snakes (Squamata: Serpentes). PLOS ONE.

[ref-75] Zaher H, Prudente ALC (2003). Hemipenes of *Siphlophis* (Serpentes, Xenodontinae) and techniques of hemipenial preparation in snakes: a response to Dowling. Herpetological Review.

