# Supplementary material for: A morphological and molecular study of Hydrodynastes gigas (Serpentes, Dipsadidae), a widespread species from South America
Source: PeerJ. 2020 Nov 25;8:e10073. doi: 10.7717/peerj.10073 (PMC7698695; doi:10.7717/peerj.10073)
Supplement: Appendix S1 — * prepared hemipenes. [file peerj-08-10073-s001.docx]

**Appendix 1.** Specimens examined. * prepared hemipenes.

***Hydrodynastes gigas*** (*n* = 144). **Argentina**: SANTA FE: *Santa Fe de la Vera Cruz*: MCN 8374; *Ogilvie*: MZUSP 10941, MZUSP 10942. **Brazil**: no geographic information: IB 87230. AMAPÁ: Tumucumaque National Park, Anacú River: IEPA 055, IEPA 155*; *Amapá*: CHUNB 15159, CHUNB 22054*; *Mazagão*: CHUNB 56729*. AMAZONAS: *Boca do Acre*: CHUNB 59249, CHUNB 59250; *Humaitá*: Puruzinho: MZUSP 5910; *Uarini*: MPEG 18837. MARANHÃO: *Arari*: MPEG 25438*. MATO GROSSO: *Cuiabá*: MCP 1748, UFMT 2311; *Nossa Senhora do Livramento*: CHUNB 65028, CHUNB 65785, UFMT 8468, UFMT 9076; *Poconé*: MCP 2439, MCP 2452, MCP 2472, MHNCI 9465, MZUSP 7309, UFMT 0019, UFMT 0026, UFMT 110; *Várzea Grande*: UFMT 4038, UFMT 5836, UFMT 8082. MATO GROSSO DO SUL: *Anastácio*: ZUFMS-REP 01587, ZUFMS-REP 02392*; *Anaurilândia*: MZUSP 10622; *Aquidauana*: IBSP 13638, MZUSP 10195–10196; ZUFMS-REP 00332, ZUFMS-REP 00335–00337*; ZUFMS-REP 01603, ZUFMS-REP 01907–01908*, ZUFMS-REP 01909–01910*, ZUFMS-REP 2383; *Bataguassu*: MZUSP 10623*; *Batayporã*: MHNCI 6365; *Corumbá*: MHNCI 7877; MHNCI 7890, MHNCI 12202; UFMT 1388, ZUFMS-REP 2393; ZUFMS-REP 01390, ZUFMS-REP 02184; *Miranda*: MZUSP 8255, ZUFMS-REP 2395; *Porto Murtinho*: ZUFMS-REP 02172; *Rio Brilhante*: MZUSP 10159. MINAS GERAIS: *Fronteira*: ZUFMS-REP 02389*. PARÁ: *Almeirim*: MPEG 16356, MPEG 16358–16359, MPEG 16361; *Cachoeira do Ariri*: MPEG 18012, MPEG 18184; *Canaã dos Carajás*: FUNED 02356; *Chaves*: MPEG 18673–18674; *Juruti*: MPEG 22651–MPEG 22652; *Melgaço*: MPEG 20018, MPEG 20945, MPEG 21864–MPEG 21868, MPEG 22141; *Muaná*: MPEG 21487; *Portel*: MPEG 20946; *Santa Cruz do Arari*: MPEG 19636; *Santarém*: MCP 7923, MCP 10104*, MCP 10604, MCP 11084*, MCP 11395, MCP 11750–11750, MCP 16942*; *Soure*: MPEG 18046, MPEG 18070–18071*. PARAÍBA: *No specific locality*: CHUFPB14837; *João Pessoa*: CHUFPB 4611, CHUFPB 4613–4614, MZUSP 8985; *Mamanguape*: CHUFPB4612. PARANÁ: *Diamante do Norte*: MHNCI 8022; *Guaíra*: MHNCI 2777. PIAUÍ: *Ilha Grande*: CZDP 0077, CZDP 0158. RIO GRANDE DO SUL: *Dilermando de Aguiar*: UFSM 0502; *Itaqui*: UFSM 01937*, ZUFSM 01995*, ZUFSM 2291; *Maçambará*: ZUFSM 3108; *Uruguaiana*: MCN 14156, MCP 6465–6467*. RONDÔNIA: *Costa Marques*: MCP 8713; *Porto Velho*: CHUNB 66534, MPEG 24223, MPEG 26059–26060, MZUSP 18571, MZUSP 18572*, MZUSP 18573, MZUSP 19566, MZUSP 19710, MZUSP 20449, MZUSP 20580, MZUSP 20716, MZUSP 21158–21159. RORAIMA: *Boa Vista*: MZUSP 9125. SÃO PAULO: *Emas*: MZUSP 2449, MZUSP 5620; *Ilha Solteira*: IBSP 36999, IBSP 37132, ZUFMS-REP 2394; *Luís Antônio*: IBSP 82902; *Marília*: MHNCI 908; *Mogi Guaçu*: MCP 769; *Pereira Barreto*: MHNCI 4511*; *Pitangueiras*: MZUSP 2719; *Santa Rita do Passo a Quatro*: MCP 643; *Santa Rosa de Viterbo*: ZUEC 2641; TOCANTINS: Porto Nacional: MZCEULP 1094; *Palmas*: CHUNB 22053, CHUNB 22068.

***Hydrodynastes melanogigas*** (*n* = 41). **Brazil**: MARANHÃO: *Carolina*: MPEG 24383–24384, MZUSP 19567–19557. TOCANTINS: Lajeado: MZUSP 15679–15680; *Palmas*: CHUNB 12802*, MNRJ 15101, MZCEULP 330, MZCEULP 461, MZCEULP 471, MZCEULP 516, MZCEULP 864, MZCEULP 896, MZCEULP 934–935, MZCEULP 938–939, MZCEULP 992, MZCEULP 1019–1021, MZCEULP 1046, MZCEULP 1087–1088, MZCEULP 1197–1198, MZCEULP 1218, MZCEULP 1264–1266, MZCEULP 1272–1273, MZCEULP 1297, MZCEULP 1358, MZCEULP 1370, ZUFMS-REP 03446. *Porto Nacional*: IBSP 65978, IBSP 66387, MZCEULP 460, MZCEULP 1258.
